# Gene Expression, Protein Function and Pathways of *Arabidopsis thaliana* Responding to Silver Nanoparticles in Comparison to Silver Ions, Cold, Salt, Drought, and Heat

**DOI:** 10.3390/nano5020436

**Published:** 2015-03-27

**Authors:** Eisa Kohan-Baghkheirati, Jane Geisler-Lee

**Affiliations:** 1 Department of Plant Biology, Southern Illinois University Carbondale, Carbondale, IL 62901, USA; E-Mail: eisa_kohan@yahoo.com; 2 Department of Biology, Golestan University, Gorgan 49138-15739, Iran

**Keywords:** silver nanoparticles, silver ions, abiotic stresses, gene expression, protein functions, pathways, *Arabidopsis thaliana*

## Abstract

Silver nanoparticles (AgNPs) have been widely used in industry due to their unique physical and chemical properties. However, AgNPs have caused environmental concerns. To understand the risks of AgNPs, *Arabidopsis* microarray data for AgNP, Ag^+^, cold, salt, heat and drought stresses were analyzed. Up- and down-regulated genes of more than two-fold expression change were compared, while the encoded proteins of shared and unique genes between stresses were subjected to differential enrichment analyses. AgNPs affected the fewest genes (575) in the *Arabidopsis* genome, followed by Ag^+^ (1010), heat (1374), drought (1435), salt (4133) and cold (6536). More genes were up-regulated than down-regulated in AgNPs and Ag^+^ (438 and 780, respectively) while cold down-regulated the most genes (4022). Responses to AgNPs were more similar to those of Ag^+^ (464 shared genes), cold (202), and salt (163) than to drought (50) or heat (30); the genes in the first four stresses were enriched with 32 PFAM domains and 44 InterPro protein classes. Moreover, 111 genes were unique in AgNPs and they were enriched in three biological functions: response to fungal infection, anion transport, and cell wall/plasma membrane related. Despite shared similarity to Ag^+^, cold and salt stresses, AgNPs are a new stressor to *Arabidopsis*.

## 1. Introduction

Nanoparticles of 1–100 nm in size [1,2] have been used in different sectors of industry [3]. In 2010, it was reported that 63%–91% of the 260,000–309,000 metric tons of worldwide products containing nanoparticles ended up in landfills while 8%–28% of them went into soil [4]. Of all nanoparticles, silver nanoparticles (AgNPs) have wide and successful applications in clothing, coatings on domestic products, food packaging, pesticides, electronics, photonics, medical drug delivery and biological tagging medicine [5,6,7,8,9,10].

Human health, food safety and environmental impacts are of prime concern regarding the usage of AgNPs [11,12,13,14]. A recent study showed that application of sewage biosolid with a low concentration of 21 ± 17 nm AgNPs (0.14 mg Ag kg^−1^ soil) to a field produced only one third of the original biomass in plants and soil microbes [15]. If AgNPs are released to the environment, they can be taken up and internalized into cells, tissues and systems. AgNPs in human, plant and microbial cells can result in adverse effects, including oxidative stress (imbalance between free radicals and their containments), cytotoxicity and genotoxicity (ability to damage the genetic information within a cell) [14,16,17,18].

AgNPs are a novel abiotic stressor and an emerging environmental contaminant to plants [19,20,21]. Uptake and accumulation of AgNPs in root caps and columella cells and transport of AgNPs through intercellular space (*i.e*., short distance transport) and via vascular tissue (*i.e*., long distance transport) were reported in *Arabidopsis thaliana* (herein, *Arabidopsis*) [19,22,23,24]. AgNPs accumulate in the cell walls of *Arabidopsis* and rice (*Oryza sativa* L.) [19,25]. Exposure of roots to AgNPs produced conflicting results, either inhibiting or promoting root growth [26,27]. But a recent study of the effects of AgNPs noted that lateral root initiation and development was promoted after the primary root apical meristem was abolished and the primary root growth was inhibited [22].

The causes of silver nanotoxicity are still in debate. One school of thoughts is that silver ions (Ag^+^) are released by AgNPs, causing chemical damage [28,29], while the other school considers the nano size AgNPs cause physical/mechanical damage [19]. Chemical silver specification in plant physiology due to physical nano silver uptake in plant tissue is also considered [30,31]. For example, ethylene is a plant hormone in various stress responses that involve Ag^+^. In the presence of such ethylene biosynthesis inhibitors, such as Ag^+^ (as silver thiosulfate, [Ag(S_2_O_3_)_2_]^3−^), in the hydroponic nutrient solution, the Fe-deficiency stress responses were inhibited in the roots of cucumber (*Cucumis sativus L. cv Ashley*) [32]. Within plant cells, more AgNPs will pose more physical harm while greater surface area of AgNPs will release more Ag^+^ to drive more toxicity. However, a recent expression study in *Arabidopsis* showed that gene expression profiles in AgNP and Ag^+^ treatments are shared and thus, concluded phytotoxicity (toxicity to plants) between the two stresses are similar [29].

Plants, being sessile, have adapted to abiotic stresses such as cold, salt, drought and heat. Cellular and molecular responses of plants to these four abiotic stresses have been studied extensively [33,34,35]. The initial responses to abiotic stresses include a transient increase of cytoplasmic Ca^2+^, elevated intracellular secondary messengers, such as inositol polyphosphate, reactive oxygen species (ROS, such as oxygen ions and peroxides) and Abscisic acid (ABA, a plant hormone), and increase in mitogen-activated protein kinase (MAPK) pathways [36,37,38,39,40,41]. The next level of stress response involves regulatory proteins that are directly involved in protection from cellular damage, and up- and down-regulation of stress-specific genes [42,43]. Secondary metabolites are also important for plants in response to abiotic stress. They are involved in structure stabilization, photoprotection, protection from antioxidants and antiradicals, signal transducing, and accumulation of polyamines; some are precursors of plant hormones and contribute to signal transduction of hormones [44,45,46,47].

When exposed to abiotic and biotic stresses, plant cell wall is the first mechanical layer of stress perception and plays a dynamic and structural role in plant adaptation [48]. Extracellular peroxidases act as modifiers of cell wall and produce superoxide, hydrogen peroxidase and oxidative burst when encountering stresses [49,50,51]. Oxidative burst triggers production of ROS, accumulation of phenylpropanoid (a type of secondary metabolites) biosynthesis enzymes, and changes of gene expression in plant defense response [50,52]. Plasmodesmata are pores of 50–60 nm in diameter and connect adjacent neighboring plant cells. Plasmodesmata can carry out trafficking and transport of proteins, mRNAs and small molecules between cells [53]. When plants are in stress, small RNAs are found in plasmodesmata [54,55]. AgNPs were found to aggregate in the cell walls and plasmodesmata in *Arabidopsis* [19] and gold nanoparticles were found to transport through plasmodesmata in poplar [56].

In contrast to commonly known abiotic stresses, the understanding of AgNP stress or silver nanotoxicity in plants is still in its infancy and remains elusive [12,15,19,31]. This study aimed to understand whether AgNP stress is similar to other abiotic stresses in plants. Four well-studied abiotic stresses (cold, salt, drought, heat) and silver ion (Ag^+^) stress were comprehensively compared with AgNP stress in *Arabidopsis*. Gene expression, protein function and pathways were used to elucidate similarities and differences in the six abiotic stresses.

## 2. Results

### 2.1. Overview of the Affected Genes by the Six Abiotic Stresses

Six sets of publically available microarray data from GEO and Array Express were used. Based on *M*-values generated from these collective data, the genes with either *M* ≥ 1 or *M* ≤ −1 were listed separately for the six abiotic stresses (Table S1). The list of differentially expressed genes showed that different number of genes in the *Arabidopsis thaliana* genome were affected by the six different abiotic stresses: between 575 and 6536 genes were differentially expressed, with AgNPs and Ag^+^ having the least (575 and 1010, respectively) and cold and salt stresses having the most (6536 and 4133, respectively) numbers of affected genes (Table 1). Drought and heat stresses have similar numbers of affected genes (1435 and 1374, respectively) (Table 1). In addition, cold stress changed the expression of 23.84% of genes (total 6536) in the *Arabidopsis* genome (27416 protein-coding nuclear genes based on the TAIR 10 release) and exhibited a predominantly down-regulating effect on gene expression. In terms of gene numbers in the AgNP, Ag^+^ and drought stresses, there were more up-regulated than down-regulated genes. The salt and heat stresses had approximately similar numbers of up- and down-regulated genes. The total number of genes affected by Ag^+^ (1010) is more than that by AgNPs (575); however both stresses induced more genes than they suppressed by a 3:1 ratio.

**Table 1:** The number list of differentially expressed genes that have more than two-fold differences (*i.e*., *M* ≥ 1 or *M* ≤ −1) in *Arabidopsis thaliana* affected by six abiotic stresses, silver nanoparticles (AgNPs), silver ions (Ag^+^), cold, salt, drought and heat. % ^a^ = regulated gene number/total affected genes.

Stress	Number of up regulated genes (% ^a^)	Number of down regulated genes (% ^a^)	Number of total affected genes	Percentage of total affected genes in genome
**AgNPs**	439 (76.34)	136 (23.65)	575	2.10
**Ag^+^**	780 (77.22)	230 (22.77)	1010	3.68
**Cold**	2514 (38.46)	4022 (61.54)	6536	23.84
**Salt**	2057 (49.77)	2076 (50.23)	4133	15.08
**Drought**	814 (56.72)	621 (43.28)	1435	5.23
**Heat**	694 (50.50)	680 (49.50)	1374	5.01

**Figure 1:**
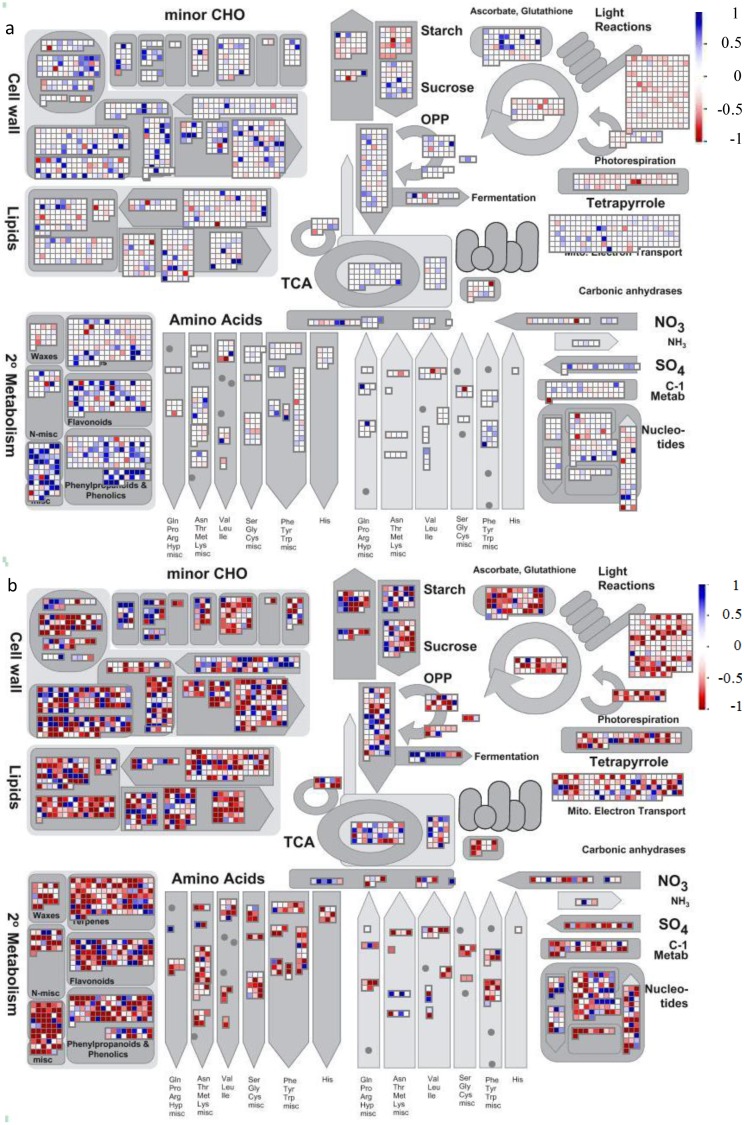

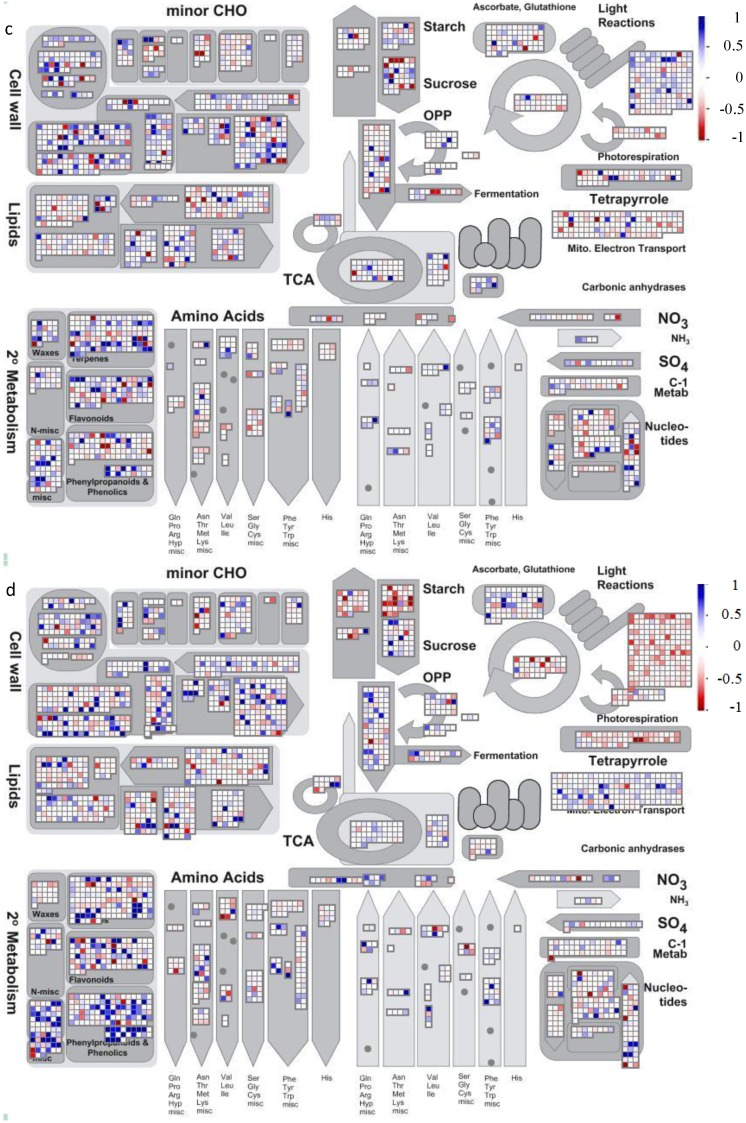

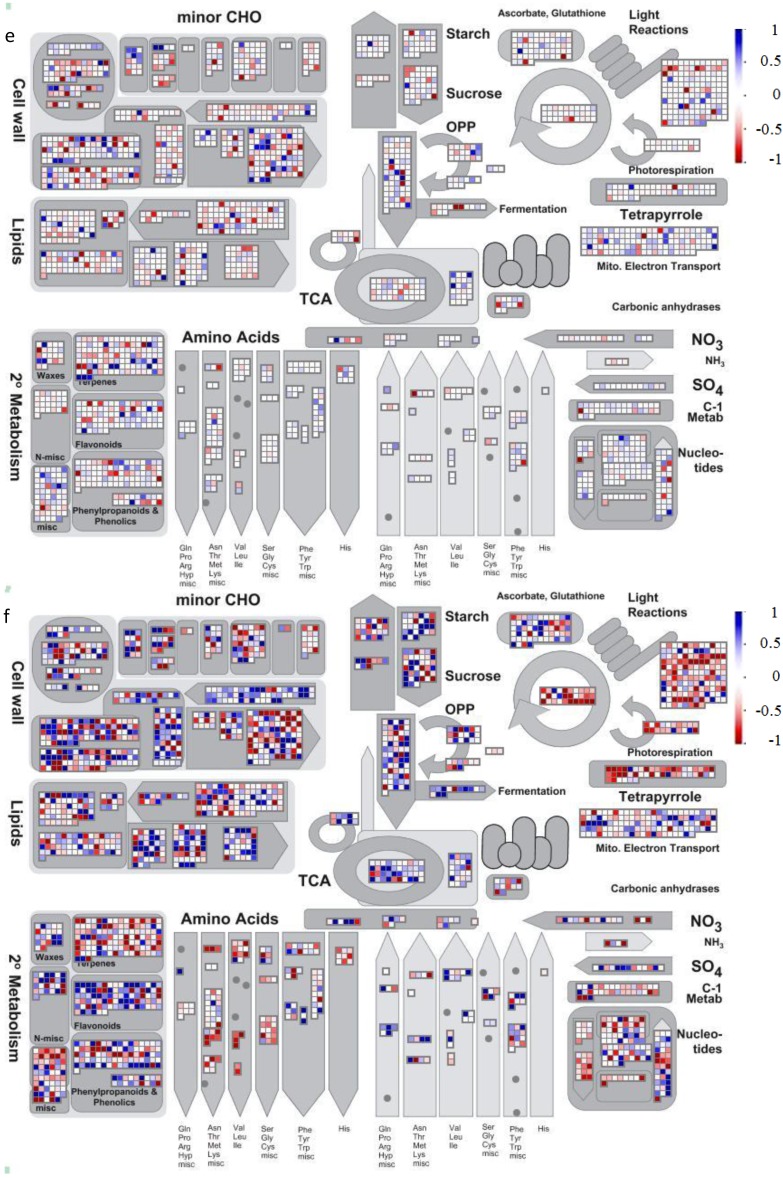
Metabolic pathway overviews for all the six abiotic stresses. *M*-value data in Table S1 for all the identified *Arabidopsis* genes were used to display in MapMan Image Annotator. Two color scale schemes were used; blue was to denote genes that were induced and red was to denote genes that were suppressed by (**a**) AgNPs; (**b**) Cold; (**c**) Drought; (**d**) Ag^+^ (AgNO_3_); (**e**) Heat; and (**f**) Salt.

Overviews of metabolic/regulatory pathway and cellular compartments were displayed for all the expressed genes in the six abiotic stresses in Figure 1. The displays allowed the first glimpse of global comparison among the six abiotic stresses: no stresses shared identical expression patterns. In the six stresses, cold stress mainly suppressed the genes in major primary and secondary metabolism (Figure 1B); salt induced the genes in both primary and secondary metabolism (Figure 1F). Drought and heat stresses showed differential patterns though shared some similarity (Figure 1C,E); heat also induced more genes than drought in both primary and secondary metabolism. AgNP and Ag^+^ stresses exhibited a similar, but not identical, pattern (Figure 1A,D). Moreover, Ag^+^ suppressed more genes in photosynthesis and sugar metabolism than AgNPs did, while AgNPs induced more genes in cell wall biosynthesis than Ag^+^.

More than 30 metabolic/regulatory pathways and cell compartments were compared to further understand the differences and similarities in the differential gene expression patterns between AgNP and Ag^+^ stresses (Figure S1). Reactive oxygen species (ROS) associated genes were up-regulated by both AgNPs and Ag^+^; this agreed with previous results [57,58,59,60]. Although it has been reported that DNA repair might be involved in the AgNP stress in animal and human cell culture studies [61,62,63,64], there was no difference in this *Arabidopsis* study (Figure S1). In the secondary metabolism, AgNPs demonstrated more up-regulated genes of lignin and lignans than Ag^+^. In nitrogen metabolism, nitrate reductase gene was up-regulated in the Ag^+^ stress; this was probably due to the source of NO_3_^−^ from AgNO_3_. Some ion transport genes were up-regulated in the AgNP stress but not present in Ag^+^ as shown in transport overview (Figure S1). The genes of sulfate (SO_4_^2−^) carbonic anhydrase pathway were up-regulated (*i.e*., induced) by AgNPs but not by Ag^+^ (Figure S1).

### 2.2. Gene Ontology Term Enrichment

**Figure 2:**
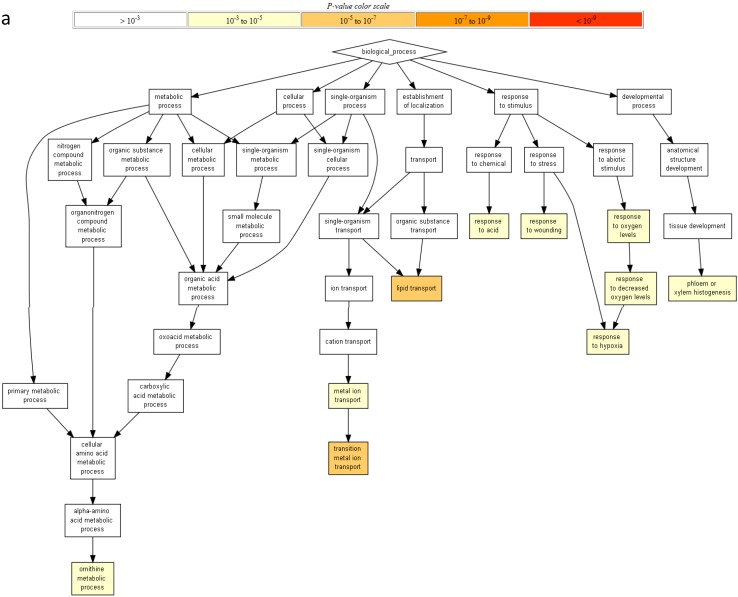

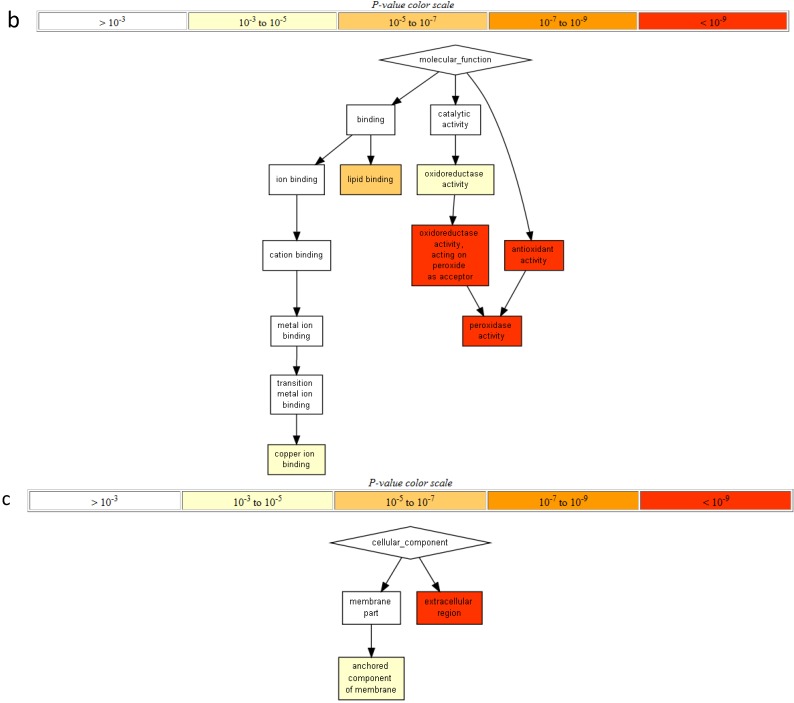
Gene Ontology (GO) term enrichment based on GO terms for total up-regulated genes by AgNP and Ag^+^ stresses. GO term enrichment results for (**a**) Biological processes; (**b**) Molecular function; and (**c**) Cellular components were presented. All colored boxes are enriched with *q*-value (FDR) less than 0.05 (*q* < 0.05) and the density of color shows the degree of enrichment, *i.e.*, red (*p*-value < 10^−9^), dark orange (*p*-value 10^−7^ to 10^−9^), orange (*p*-value 10^−5^ to 10^−7^), yellow (*p*-value 10^−3^ to 10^−5^) and white (*p*-value > 10^−3^).

No difference was found in gene ontology (GO) term enrichments of the total up-regulated genes by AgNP and Ag^+^ stresses (Figure 2). In addition, there was no enrichment for the down-regulated genes by AgNP and/or Ag^+^ stresses. The up-regulated genes in both stresses were enriched in lipid transport and transition metal ion in the category of biological process, peroxidase activity in the category of molecular function, and extracellular region in the category of cellular component (Figure 2A–C; Table S2).

**Figure 3:**
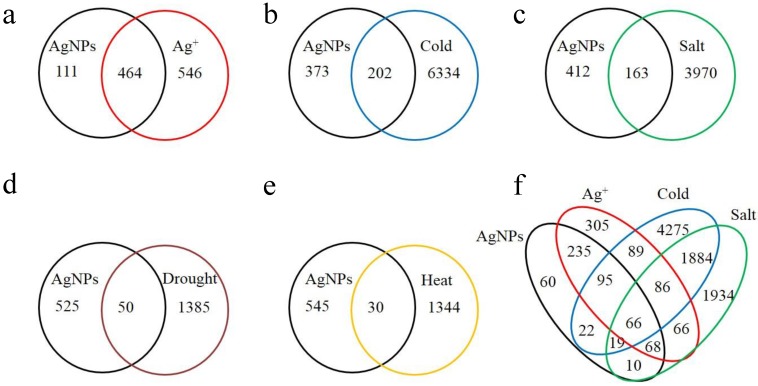
Venn diagrams of the genes with more than two fold expression changes and shared among the six stresses. (**a**–**e**) were two way comparison and (**f**) was four way. Overlapped areas were shared genes while non-overlapped areas were specific/unique genes for individual stress. (**a**) Between AgNPs and Ag^+^; (**b**) Between AgNPs and cold; (**c**) Between AgNPs and salt; (**d**) Between AgNPs and drought; (**e**) Between AgNPs and heat; (**f**) Among AgNPs, Ag^+^, cold and salt.

To understand the similarities in AgNP and Ag^+^ stresses, GO term enrichment analysis of the shared genes in both the stresses was compared. The Venn diagram data showed a total of 464 genes were shared by AgNP and Ag^+^ stresses (Figure 3A; these genes are listed in Table S3). These genes were enriched in lipid transport (GO:0006869) and transition metal ion transport II (GO:0000041) in the category of biological process (Figure S2A); antioxidant activity (GO:0016684) and peroxidase activity (GO:0004601) in the category of molecular function (Figure S2B); the extracellular regions in the category of cellular components (GO:0005576) (Figure S2C).

To understand the differences in AgNP and Ag^+^ stresses, GO enrichment analysis was compared for the specific genes in either AgNP or Ag^+^ stress. A total of 546 Ag^+^-specific genes (Figure 3A, listed in Table S3) were enriched for more than 30 biological processes (Figure S3A). For example, nitrate transport (GO:0015706), transition metal ion transport I (GO:0000041), response to nitrate (GO:0010167). In contrast, 111 AgNP-specific genes (Figure 3A, listed in Table S3) were slightly enriched for only one biological process, response to fungus (GO:0009620) (Figure S3B).

### 2.3. Protein Domain Enrichment

**Figure 4:**
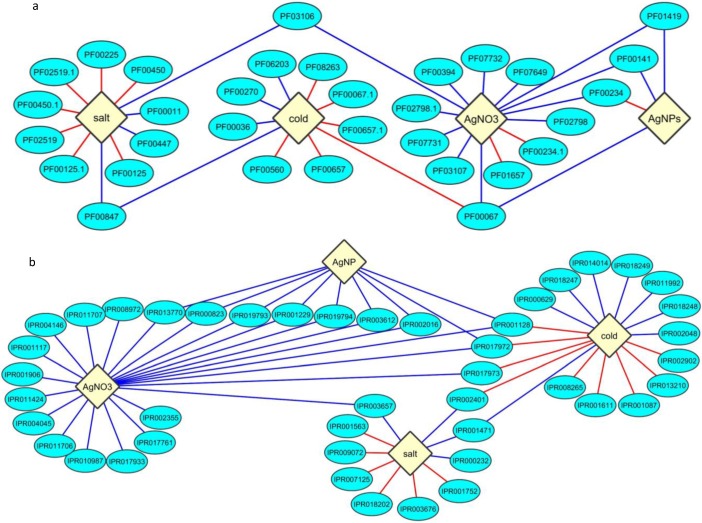

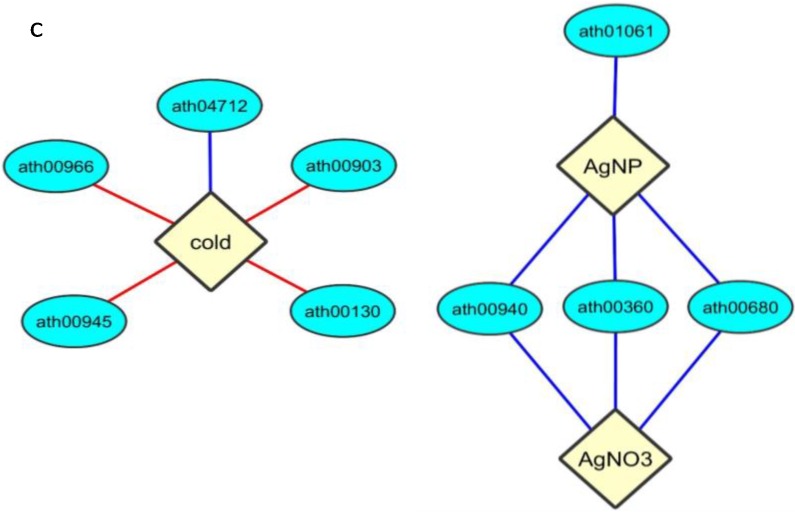
PFAM protein domain, InterPro protein class, and KEGG pathway enrichment of the genes with more than two fold expression changes for all the six abiotic stresses. (**a**) Visualization of 32 unique enriched PFAM protein domain across the six abiotic stresses; (**b**) Display of 44 definite enriched InterPro classes associated with the six stresses; (**c**) Nine enriched KEGG pathways in six stresses were shown. The enrichment results were visualized using Cytoscape 3.1.0, where blue edges denote enrichment for up-regulated genes and red edges denote enrichment for down-regulated genes. The description of PFAM protein domain, InterPro protein class, and KEGG pathway were in Tables S2, S4 and S5.

Protein domains curated by PFAM are categorized based on the similarity of global sequence alignments [65,66]. The coded proteins of induced and suppressed genes by the six abiotic stresses were subjected to PFAM protein domain enrichment analysis. A total of 32 uniquely enriched PFAM protein domains were identified across the four abiotic stresses, cold, salt, AgNPs, Ag^+^ (see Table S2). This implies that these four stresses differ from the other two stresses, drought and heat. Four enriched domains, PF01419:Jacalin, PF00141:peroxidase, PF00234:Tryp_alpha_amyl and PF00067:p450, were shared in AgNP and/or Ag^+^ stress. PF01419:Jacalin, Jacalin-like lectin domain, is a mannose/galactose-binding lectin domain with three beta-sheets [67,68]. Jacalin-like lectin domain containing proteins include Jacalin, which is seed lectin and agglutinin from jackfruit (*Artocarpus heterophyllus*) [69]. The peroxidases containing PF00141:peroxidase domain use hydrogen peroxide (H_2_O_2_) to accept electrons and produce water when catalyzing oxidative reactions [70]. One class of plant-specific peroxidases is involved in tissue-specific reactions; two of their notable reactions are ethylene production and defense against wounding [71]. The proteins containing PF00234:Tryp_alpha_amyl domain is a group of plant lipid transfer proteins (LTPs) and is involved in plant defense mechanisms [72,73]. LTPs transfer lipids in membranes. The proteins containing the PF00067:p450 domain belong to a superfamily of cytochrome p450 (p450), which catalyze the final reactions [RH + O_2_ + NADPH + H^+^ → ROH + H_2_O + NADP^+^] in biological electron transfer chains [74]. Plant p450s are involved in diverse reactions, especially in plant defense and secondary metabolite production [75,76,77]. Among these four enriched domains, the genes to encode the proteins containing PF00067:p450 domains were also associated with down-regulated genes by cold [78,79]. In addition, PF03106:WRKY and PF00847:AP2 were shared by the upregulated protein-encoding genes in Ag^+^ and salt stresses and in salt and cold stresses, respectively. PF03106:WRKY domains belong to DNA-binding transcription factors which are one of the largest signaling/regulatory protein families in plants [80,81]. WRKYs could integrate with such signaling cascades as mitogen-activated protein kinase (MAPK), MAPK kinases and defense proteins. The proteins containing the PF00847:AP2 domain are transcription factors Apetala 2 in the large family of AP2/EREBP [82]. EREBP is ethylene-responsive element binding protein. It implied that the signaling pathways in Ag^+^ and salt stresses are involved in ethylene and WRKY transcription factors. The 32 enriched protein domains with their related stresses could be visualized in Cytoscape in Figure 4A.

### 2.4. Enrichment of InterPro Protein Classes

InterPro [83,84] classifies proteins into families and predicts domains and reaction sites by providing functional analysis of proteins [85,86]. InterPro classified protein (herein, InterPro protein class) enrichment was based on predictive models as protein signatures, which were annotated in the InterPro database. There would be a similarity between PFAM protein domain analysis and InterPro protein class analysis; but the enrichment analysis by the latter could provide more specific data about interested proteins, due to protein signatures. No enriched InterPro protein classes were found related to drought and heat; this result matched PFAM protein domain enrichment (Tables S2 and S4). Among the four abiotic stresses studied (cold, salt, AgNPs, Ag^+^), forty-four definite InterPro protein classes were found associated with one or multiple stresses (Figure 4B and Table S4). Both the enrichment analyses of PFAM protein domains and InterPro protein classes demonstrated that AgNP stress induced more peroxidase (including domain, signature and function) encoding genes than Ag^+^ stress did. For example, IPR000823:Plant peroxidase; IPR002016:Heme peroxidase, plant/fungal/bacterial; IPR019794:Peroxidase, active site; PF00141:peroxidase in PFAM protein domain.

Between PFAM domain and InterPro protein class enrichment analyses, as predicted, an overall similarity was found in the four abiotic stresses (AgNPs, Ag^+^, cold, salt; Figure 4A,B). However, there were two major differences in these two enrichment analyses. The first difference was differential occurrences of p450 domain-containing proteins in the four stresses (Figure 4B and Table S4). Based on the PFAM domain enrichment (Figure 4A), only PF00067:p450 domain was associated with the up-regulated genes by cold, and the down-regulated genes by AgNPs and/or Ag^+^. But the InterPro protein class enrichment presented differential results in four different classes of p450s. IPR002401:Cytochrome p450 (E-class, group I) was shared between the up-regulated genes by salt stress and down-regulated genes by cold. IPR017973:Cytochrome p450 (*C*-terminal region) was shared between the up-regulated genes by Ag^+^ and the down-regulated genes by cold. Two other InterPro protein classes, IPR001128:Cytochrome p450 and IPR017972:Cytochrome p450 conserved site, were shared between the up-regulated genes by cold and down-regulated genes by AgNPs and/or Ag^+^ (Figure 4B and Table S4).

The second difference in the two protein enrichment analyses was lipid transfer proteins (LTPs) (Figure 4A,B). LTPs shuttle phospholipids and other fatty acid groups between cell membranes to build cell walls [72]. Phospholipids are major components in cell membrane, including inositol phosphate (InoP). Despite the fact that several LTPs were shown in both the enrichment analyses, PF00234 was only in the PFAM protein domain analysis but was not in the InterPro protein class analysis (Figure 4A,B). PF00234 is protease inhibitor/seed storage/LTP family domain [87].

### 2.5. Enrichment within KEGG Pathways

Kyoto Encyclopedia of Genes and Genomes (KEGG) annotation was used to show biological pathway enrichment of up- and down-regulated genes of the six abiotic stresses. The connectivity of each pathway related to the six stresses studied was displayed in Figure 4C and listed in Table S5. Nine unique KEGG pathways in *Arabidopsis* (*i.e*., prefix with “ath”) were found in differentially expressed genes induced by AgNP, Ag^+^ and cold stresses; but no enriched KEGG pathway was found by salt, drought, and heat stresses. These nine KEGG pathways were characterized into three groups, (1) five for cold stress; (2) three for both AgNP and Ag^+^ stresses; and (3) one for only AgNPs, which was ath01061:Biosynthesis of phenylpropanoids.

The three shared KEGG pathways between AgNP and Ag^+^ stresses-regulated genes were in secondary metabolism and methane metabolism. Ath00360:Phenylalanine metabolism is involved in metabolism of terpenoids and polyketides. The pathway of Ath00940:Phenylpropanoid biosynthesis starts with phenylalanine and produces a variety of secondary metabolites as precursors for signaling (such as phenolic volatiles, coumarin, flavonoids) and structure (such as lignin, suberin, wall-bound phenolics) [88,89]. Ath00680:Methane metabolism can reduce NADP^+^ to NADPH and convert glycine to serine.

The single KEGG pathway of ath01061:Biosynthesis of phenylpropanoids was enriched in the only AgNP up-regulated genes. The ath01061 pathway starts with the products of primary metabolism (*i.e*., glycolysis and the tricarboxylic acid cycle/the Krebs cycle) and ends up phenylpropanoids [88,89,90,91]. Phenylpropanoids are precursors to diverse secondary metabolites, such as tannins, lignans and flavonoids.

### 2.6. Comparison of Shared and Specific Genes among Six Abiotic Stresses

Figure 3 showed that the number of the shared genes between two stresses (AgNP *vs.* Ag^+^, AgNP *vs.* cold, AgNP *vs.* salt, AgNP *vs.* drought, AgNP *vs.* heat) and among four stresses (cold, salt, AgNP and Ag^+^). The gene number shared between AgNP and Ag^+^ stresses (464) was much higher than those between AgNP and with the other four stresses (202, 163, 50, and 30, respectively). The high number of shared genes might partially attribute to the potential release of silver ion (Ag^+^) from AgNPs [29,92]. Nevertheless, 111 genes were AgNP-specific but not Ag^+^-specific (Figure 3A). This may be in agreement with our previous publication that indicated the effects of AgNPs were different from Ag^+^ [19,31].

Among the other four abiotic stresses (cold, salt, drought, heat) studied, AgNP stress shared the most genes affected with cold, followed by salt, then drought and finally, heat (Figure 3B–E). In the category of biological processes, gene ontology (GO) term enrichment for AgNP-cold shared genes were involved in response to acid (GO:0001101), and in response to oxygen containing compounds (GO:1901700); in the category of molecular functions, involved in catalytic activity; and in the category of cellular components, involved in extracellular region (Figure S4). Based on the GO enrichment analysis, the similarity of AgNP and cold stresses may be due to their mechanical damages on membrane/cell wall and induction of oxidative stress [93,94,95]. The four-way Venn diagram showed that sixty-six genes were shared in response to AgNP, Ag^+^, cold and salt stresses. These 66 genes were enriched in response to oxygen containing compounds and regulation of reactive oxygen species (ROS) metabolism processes (GO:2000377) (Figure S5). Shared genes across three, four or six different stresses were also provided in Figure S6 and Table S6.

There were another 60 genes specific to only AgNP stress but not to Ag^+^, cold, salt, and even to drought and heat (Table S7). These genes were enriched in ion transport process, especially anion transport (GO:0006820). This implies that *Arabidopsis* plants in the AgNP stress may have utilized anion transporters to maintain ion homeostasis (or charge equilibrium) from unknown mechanism(s) induced by AgNPs. The release of Ag^+^ by AgNPs cannot explain this phenomenon.

Only four genes (At5g10040, At4g17470, At1g01130, and At1g69500) were shared by all the six abiotic stresses—AgNP, Ag^+^, cold, salt, drought, and heat stresses Table S6). At5g10040 encodes one unknown protein involved in anaerobic respiration; At1g01130 one unknown calcium/calmodulin-dependent protein kinase-like [96]; At1g69500 cytochrome p450 [97]; and At4g17470 alpha/beta-hydrolases superfamily protein involved in changes in the endoplasmic reticulum lipid properties when experiencing low temperature [72].

### 2.7. Protein-Protein Interaction Networks of Affected Genes by Six Abiotic Stresses

**Figure 5:**
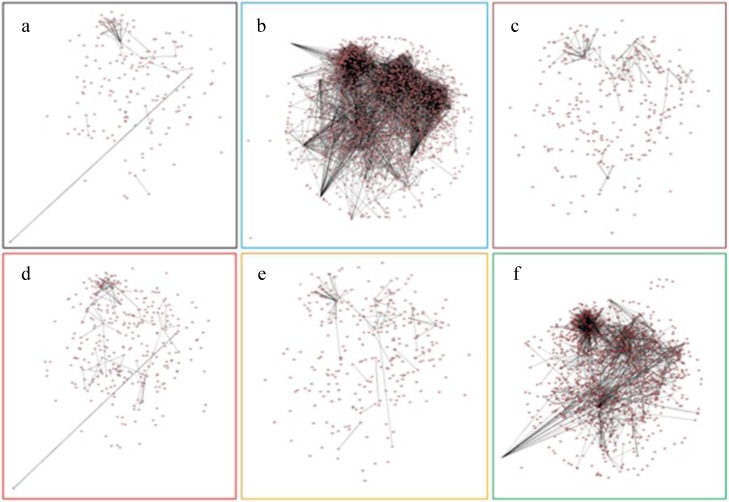
Protein-protein interaction (PPi) networks of affected genes (*i.e*., *M*-value ≥ 1 or ≤ −1) for all the six stresses. (**a**) AgNPs; (**b**) Cold; (**c**) Drought; (**d**) Ag^+^; (**e**) Heat; (**f**) Salt. Nodes represented proteins and edges showed the interaction between proteins.

The protein-protein interaction (PPi) networks of the affected encoding genes (*i.e*., *M*-value ≥ 1 or ≤ −1) for all the six stresses were created (Figure 5). The PPi network of the cold stress was most densely connected, followed by that of the salt stress. The other four stresses showed sparsely connected with few protein hubs. The PPi network of the cold stress included 6536 gene-encoded proteins and they could build the biggest network (among the six stresses) with an average connectivity of 1.94269 (Table S8). An average connectivity of more than 1 indicates that the number of edges (*i.e*., interactions) is more than the number of nodes in the network. This means each protein has averaged more than one connection with other proteins.

A PPi network was also created for shared and specific gene-encoded proteins that were induced/suppressed by the stress of AgNPs and/or Ag^+^ (Figure S7A; Table S9). This network contained 368 nodes (derived from 1127 AgNP and/or Ag^+^ affected genes) and 129 edges. The majority of the nodes (70%) had no connectivity. But in the AgNP stress, there was a major hub of heptahelical transmembrane protein (HHP2; encoded by At4g30850) (Figure S7B). HHP2 was reported to be involved in membrane transport [98,99]. At4g30850 gene was up-regulated only by the AgNP stress but not by the other five stresses. Interestingly, the hub of HHP2 had 20 edges (*i.e*., interactions) with Ag^+^-stress specific gene-encoded proteins and 11 edges with AgNP and Ag^+^ shared gene-encoded proteins. Some of the edges include such transporters as ABC transporter family proteins, oligopeptides transporter, nucleotide/sugar transporter family protein and copper transporter. The list of HHP2 connected nodes and their connectivity’s were presented in Table S10.

## 3. Discussion

### 3.1. Similarities and Differences of AgNP Stress and Five Other Abiotic Stresses

Plants respond to abiotic and biotic stresses by changing their gene expression and metabolism in order to adapt the stresses [100,101]. *Arabidopsis* plants responded to cold and salt stresses by changing expression of large numbers of genes, 23.84% and 15.08% of their genome; however, AgNP stress did by only 2.10%, the lowest of all stresses examined (Table 1). It implies *Arabidopsis* plants has a much reduced response to AgNP stress by up-regulating/down-regulating fewer genes and producing/decreasing less of their encoded products than the other five stresses (Ag^+^, cold, salt, drought and heat). This indicates that AgNPs are a new different stressor for *Arabidopsis* plants and in different plants and crop species [19,26,31,102]. However, the genetic differences elucidated in this study could be qualitative results that cannot be statistically evaluated nor in consideration of gene interactions.

Some of abiotic and biotic stresses trigger reaction oxygen species (ROS) responses [36,37,103]. The ROS reaction cascade triggered by stresses occurs in the membrane of the plant cells by generation of such secondary messengers as calcium and ROS, and then follows by phosphorylation of downstream proteins. This study showed that ROS-regulated genes (shown in Figure S5) and GO:2000377 were shared by AgNP, Ag^+^, cold, and salt stresses. Although there is no direct evidence of secondary messenger calcium accumulation nor AgNP receptors found in *Arabidopsis* cell membranes, several studies reported induction of ROS in plants exposed to AgNPs [59,104]. In addition, the enrichment of antioxidant activity for the genes affected by AgNPs (Table S11) was in agreement with those studies. Upon the increase of ROS against stress, plants also produce antioxidants to remove ROS from damaging cells [38]. At the same time, ROS are also intermediate signals (*i.e.*, secondary messengers) to induce Abscisic acid (ABA) and calcium cascade [105]. ABA regulates approximate 10% of protein-coding genes in the *Arabidopsis* genome, the highest percentage among all the plant hormones [106]. Animal and human cell line studies showed generation of ROS and use of mitogen-activated protein kinase (MAPK) pathway to transduce signals of AgNPs [61,107]. Plants may also utilize oxidative stress signaling for AgNPs by using MAPK cascade modules.

### 3.2. Similarity and Difference of AgNP and Ag^+^ Stresses

This study showed no major difference in the enrichment analyses of GO term, PFAM protein domain, InterPro protein classification, and KEGG pathways of AgNP and Ag^+^ affected genes (Figure 2, Tables S2, S4, S5 and S11). However, most of their enrichments were related to oxygen level and ROS, which are also regulated by cold and salt stresses. Enrichment of ornithine metabolism process for AgNP/Ag^+^ affected genes illustrated that AgNP and Ag^+^ induced osmotic stress, which consequently changed ornithine metabolism to synthesize more osmolytes, such as polyamines and proline [108,109]. Osmotic stress is a rapid change in the solute concentration around a cell.

Another considerable enrichment in the both AgNP and Ag^+^ stresses was for phloem or xylem histogenesis. This enrichment could be related to inhibition of primary root growth by AgNPs or Ag^+^; thus, it implies possible production of lateral roots [21,22,25,27]. It was reported that AgNPs inhibited root growth by directly destroying meristematic cells (able to divide) in root apical meristem (RAM) [19] and indirectly promoted lateral root growth in *Arabidopsis* [22]. Although some lateral root primordia were destroyed by AgNPs, the others could have survived to take over the responsibility of nutrient and water uptake from primary roots [22]. Ag^+^ (of AgNO_3_) improved rooting of vanilla (*Vanilla planifolia*) explants [110]. Nevertheless, improved root growth by Ag^+^ cannot explain why *Arabidopsis* RAM was abolished by AgNPs.

On the other hand, the phytotoxicity of AgNPs has been shown to be much worse than their released Ag^+^ [19,31]. AgNPs could contribute their toxicity in both the nanoparticles themselves (*i.e*., physical nano size) and their dissolved and released Ag^+^ to their surroundings (*i.e*., chemical Ag^+^ factor) [14,19,31]. The *Arabidopsis* root phenotypes in AgNP stress differ from those in the identical concentrations of the released Ag^+^ by AgNPs. In addition, AgNPs presented size- and concentration-dependent toxicity [19,63]. Any study using only Ag^+^ (e.g., an AgNO_3_ solution) could not answer size-dependent toxicity of AgNPs.

Cationic (or positive-charged) nanoparticles can pass through cell membranes by creating transitory holes in membranes [111]. This process, thus, induces cytotoxicity. If Ag^+^ could penetrate plasma membrane fast, then cytotoxicity would be severe. Thus, it is hypothesized that fast penetration of Ag^+^ across plasma membrane could affect photosynthetic electron transport and slow down primary metabolic pathways sooner [112,113]. Once primary metabolic pathways were slowed down, affected genes would be up- and down-regulated to allow plants to adapt into their Ag^+^ stress.

GO term enrichment analysis presented unique differences between AgNP and Ag^+^ stresses (Figure S3). The genes specifically regulated by Ag^+^ were enriched for response to nitrate and related processes. This probably attributed to the addition of NO_3_^−^ (in AgNO_3_), a by-product of Ag^+^ stress. Enrichment of nitrate related metabolism pathways could be corresponding with Ag^+^ mediated responses such as in polyamine biosynthesis, ethylene- and calcium-mediated pathways [114]. PPi networks of the AgNP and Ag^+^ affected genes-encoded proteins were similar to each other (Figure 5A,D, respectively); but the network of Ag^+^ stress has slightly more connectivity than that of AgNP stress.

### 3.3. Comparison of AgNP and Cold Stresses

Cold stress changed the expression of approximately a quarter of total genes in the *Arabidopsis* genome and exhibited a predominantly suppressive effect on gene expression and most metabolic pathways (Table 1, Figure 1B). Based on the Venn diagram analysis among the four abiotic stresses (cold, drought, heat, salt) studied, AgNP stress shared the most genes affected by cold (Figure 3B), up to 35% of AgNP regulated genes were also regulated by cold. Among the genes shared by AgNP and cold stresses, 49 of them (including cold responsive gene, COR) were regulated by *DREB1A* gene-encoded protein DREB1A (dehydration responsive element binding factor 1A). DREB1A is also called CBF3 (C-repeat binding factor 3) and acts as a main regulon (a group of genes regulated by the same regulatory protein) in cold response [115,116]. Particularly in this regulon, the ICE1-CBF-COR signaling pathway has been known in regulating plant response to cold stress [117,118,119,120,121,122]. ICE is inducer of CBF expression 1. CBF (*i.e*., DREB1) acts as a major player of the *Arabidopsis* regulatory network in response to cold stress; this could imply a possible signaling crosstalk between CBF-regulated cold response pathway [123] and other non-temperature signaling transduction pathways such as AgNPs.

Membrane leakage is the primary damage to cells upon cold stress [124], while ROS results in initial signaling of cold stress [42]. Thus, cold acclimation by plants includes stabilization of cell membrane integrity, production of ROS signals and antioxidative pathways, elevated levels in sugar and osmolytes, such as polyamines [108,125]. The similar ROS signaling and antioxidant pathways have been reported in the studies of rat cortical cell cultures and human murine dendritic cell lines when treated with AgNPs [60,126]. Despite the fact that no direct evidence of AgNP entry/transport to membrane is found in plant cells, aggregation of AgNPs in vacuoles and at plasmodesmata were recently reported [19,31,127] as well as gold and carbon coated iron nanoparticles [56,128]. It indirectly implies that AgNPs, like cold stress, may induce ROS generation and consequently, change the physical state of membranes.

GO term enrichment analysis also confirmed that both cold and AgNP stresses were enriched in the molecular functions of response to ROS. In addition, both stresses were enriched in the molecular function of response to fungus. In cold stress, ice formation was reported to cause a mechanical strain on cell wall and membrane leading to cell rupture in winter wheat (*Triticum aestivum*) [129]. Rupture of cells and their cell walls might have released some oligosaccharides similar to the elicitors induced by fungal infection [130]. Moreover, PFAM domain enrichment analysis showed PF00067:p450 domain associated with AgNP and/or Ag^+^ up-regulated, and cold down-regulated genes (Table S2). It was reported that there were more than 270 cytochrome p450 genes in the *Arabidopsis* genome and they all played important roles in development and responses to abiotic and biotic stress [131]. However, most of stress-induced p450 genes could be triggered by multiple stresses but each response was regulated according to individual stress [79]. This concurred the PFAM enrichment analysis in the comparison of cold and AgNP stresses; Pf00067 was enriched for the down-regulated gene-encoded proteins in cold stress but it was enriched for the up-regulated gene-encoded proteins in AgNP stress (Figure 4A and Table S2).

### 3.4. AgNP-Specific Responses in Genes and Functions

AgNPs have been commonly used in human society for their unique antimicrobial properties [5,8]. They have been studied in assays, transport and accumulation and microarray studies to confirm their phytotoxicity (toxicity to plants) [19,29,31]. Although the controversy between AgNPs and Ag^+^ continues, this current study could provide new insights and shed light to this controversy. Despite the fact that AgNP and the other five abiotic stresses (Ag^+^, cold, drought, heat and salt) affected similar metabolic pathways, AgNPs had some unique effects on *Arabidopsis* plants. First, the gene ontology (GO) term enrichment analysis demonstrated that AgNP specific gene-encoded proteins were enriched in two biological processes; one was enriched in Response to fungus (*i.e*., enriched beta-1,3-endoglucanase domain) and the other was enriched in Anion transport. Response to fungus demonstrates a similarity of AgNPs to biotic stresses (fungal infection specifically) and wounding. Anion transport implies that the AgNP stress regulated different ion transporters from Ag^+^ or salt (Na^+^) did. Second, among all the 60 AgNP-specific genes, they could be sorted into two categories, protection from oxidative burst and involvement in cell wall and/or plasma membrane. The category of protection from oxidative burst includes glutathione S-transferase and p450s [132]. The second category was beta carbonic anhydrase 3, cellulose synthase, glycosyl hydrolase superfamily protein, alpha/beta-hydrolases, hydroxyproline-rich glycoprotein, beta glucosidase, glycosyl hydrolase, and some related to proteolysis processes such as serine carboxypeptidase-like 30.

## 4. Experimental Section

### 4.1. Microarray Data and Data Processing

Microarray data of six abiotic stresses in *Arabidopsis thaliana* were obtained from Gene Expression Omnibus (GEO) [133,134] and from Array Express in the European Molecular Biology Laboratory [135,136]. They are silver nanoparticles (herein, AgNPs), silver nitrate (AgNO_3_; herein, Ag^+^), cold, salt, drought and heat. The microarray data were listed as below.

E-MEXP-3950. AgNP and Ag^+^ stresses after 10-day treatment [29].
GSE5620.   Control after 24 h treatment [137].
GSE5621.   Cold stress after 24 h treatment [137].
GSE5623.   Salt (NaCl) stress after 24 h treatment [137].
GSE5624.   Drought stress after 24 h treatment [137].
GSE5628.   Heat stress after 24 h treatment [137].

E-MEXP-3950 data came from whole seedlings after growing for 10 days in the presence of 5mg/L AgNPs (of 20 nm) or Ag^+^ (*i.e*., AgNO_3_). Normalized log-2 transformed transcriptomic data [29] were used to find the genes with more than two fold expression changes. Three biological replicates for each treatment/control were averaged. The treatment average minus control average was taken as the final value for each gene. Since normalized log 2 transformed data, *i.e.*, *M*-values,

(1) $$\left[M-value=log_{2}\left(\frac{treatment}{control}\right)\right]$$

were used, the final values of more than 1 or less than −1 present a more than two fold change in gene expression. Genes with *M*-values ≥ 1 and *M*-value ≤ −1 mean more than two fold up-regulated and down-regulated, respectively.

The comprehensive data set at AtGenExpress [137] was used to identify genes with more than two fold expression changes under four diverse abiotic stress conditions: cold, salt, drought, heat. The AtGenExpress data came from shoots and roots separately while Kaveh’s data [29] came from whole seedlings. Thus, the former’s data would be proportionally scaled in order to be comparable with the latter’s data. In doing so, a fresh weight biomass shoot-root ratio (S/R) was utilized, based on a similar growth stage and growing in a comparable medium [138]. The formula to convert shoot and root signal to whole plant signal is:

[whole plant signal = S/R ratio * shoot signal + root signal]
(2)

Once whole plant signal was calculated for each biological replicate, *M*-value was calculated for each replicate. Next, two biological replicates (in AtGenExpress data) were averaged. An initial list of the genes with more than two fold changes in the expression of six abiotic stresses was prepared for further analyses (Table S12). Based on Table S12, Venn diagrams were also created to display numbers of genes which were shared by the six abiotic stresses and which were unique for specific stresses for further analyses.

### 4.2. Visualization of Affected Genes in Metabolic Pathways

Microarray data of the six abiotic stresses were parsed into their respective metabolic pathways and cell compartments using MapMan software [139]. MapMan (version 3.5.1R2) was employed to display microarray data of the six stresses in a variety of metabolic and signaling pathways. *M*-value data in Table S1 were used for all the *Arabidopsis* identified genes (based on TAIR10 annotation) [140]. They were displayed in MapMan Image Annotator in two color scale schemes: blue is used to denote induced genes and red to denote suppressed genes.

### 4.3. Coded Proteins of Affected Genes by the Stresses in Protein-Protein Interaction Networks

Both the *Arabidopsis* predicted interactome 2.0 [141] and an experimentally verified interactome [142] were used as reference sets, based on protein orthologues (*i.e.*, proteins from divergence of a common gene), to create a PPi network for the coded proteins of the affected genes by the six abiotic stresses. The genes with *M*-value ≥ 1 or ≤ −1 (see Table S12) were first used as a coded protein query set to search their interacting protein partners. This query set of proteins (*i.e.*, coded gene products) became an initial reference network to find their edges (*i.e*., interacting proteins). Next, these edges were used to identify protein analogues (*i.e*., proteins from convergence of different genes but of the same function) and to expand a PPi network. The set of paired proteins from the query set and their analogues was then exported as a new PPi network. The combination of the initial reference network and its expanded networks became the final PPi network of the affected gene-coded proteins from the six abiotic stresses. All the proteins in the final PPi network were displayed in the Cytoscape 3.1.0 [143].

### 4.4. Enrichment Analyses of Differentially Expressed Genes in Six Abiotic Stresses

Two web-based applications, GOrilla [144] and DAVID 6.7 [145], were used in enrichment analyses to characterize the underlying biological processes, molecular functions and cellular components for the differentially expressed genes in the six abiotic stresses (*i.e.*, Table S12). The analyses investigated the coherence of the data across different mechanisms of *Arabidopsis* responses to the six abiotic stresses. Enrichment analyses included gene ontology (GO) [146], PFAM for protein domains [66], InterPro for protein signatures and functions [83,147], and Kyoto Encyclopedia of Genes and Genomes (KEGG) pathways [148]. GO term enrichment for biological process, molecular function and cellular component were performed by GOrilla. Annotated and characterized genes in *Arabidopsis* (TAIR10) were a “background gene list”. GOrilla used a list of up- and down-regulated genes (from each stress) as a “target gene list” to search for GO enriched terms in this “target gene list” in comparison to the background gene list. PFAM domains came from global (amino acid) sequence alignment while InterPro classes came from local shorter aligned sequences (*i.e*., signatures) and catalytic sites (*i.e*., functions). *Arabidopsis* gene IDs of TAIR 10 as background list and the target gene list (Table S12) were subjected to DAVID 6.7 when enrichment analyses of PFAM domains, Interpro protein classes and KEGG pathways were performed. The output data by GOrilla and DAVID 6.7 were then filtered, using the *q*-values (*i.e*., False Discovery Rate; it was adjusted from *p*-value) less than 0.05 (*i.e.*, *q* < 0.05). *p*-value is the probability of the observed results on the null hypothesis which is true. Enrichment analyses were also done for shared and/or specific genes that were derived from Venn diagram analysis (see below) for AgNP when compared with the other five stresses.

### 4.5. Comparison of Shared and Specific Genes in Venn Diagrams

A graphical Venn diagram helps visualize complex biological data sets and illustrate the overlap in genes shared by different conditions. One calculator and drawing Venn diagram’s web-tool [149] was employed to compare genes with more than two fold expression difference (*i.e*., *M*-value ≥ 1 or ≤ −1) that were shared by the six abiotic stresses. The list of genes with more than two fold expression changes (Table S12) was uploaded to the site and output data were used to draw the diagrams. Two-way Venn diagrams were used to compare AgNP with the other five abiotic stresses. Three-, four- or six-way Venn diagrams were also used to compare shared genes across three, four or six stresses.

### 4.6. Plasmodesmata Related Genes Expressed in AgNP and Ag^+^ Stresses

Two approaches were employed to identify how many genes were related to plasmodesmata and also affected by AgNPs and/or Ag^+^. First, a search was performed using “plasmodesmata” in the gene description and GO terms of AgNP and/or Ag^+^ affected genes, which were obtained from BioMart [150]. Second, a list of genes that are directly related to plasmodesmata was prepared based on GO terms in AmiGO2 [146,151,152,153,154], GONUTS (the Gene Ontology Normal Usage Tracking System [155], and literature search [156,157,158,159,160,161,162,163,164,165,166,167,168,169,170]. Altogether, a list of the 26 plasmodesmata related genes was collected and it was provided in Table S13.

## 5. Conclusions

Despite the similarities of regulated genes by AgNP stress and five other stresses, there are distinct differences by AgNPs. There are 60 AgNP-specific genes that are not affected/regulated by the other five stresses. The shared properties of Ag^+^ and AgNP stresses were due to chemical Ag^+^ ions; but AgNP stress differed from Ag^+^ stress, probably resulting from physical/mechanical damage due to nano-size of AgNPs. The similarities of AgNP and cold stresses could result from their mechanical damages and induction of ROS; but the two stresses were different. In sum, despite the shared similarity in gene expression and metabolic pathways to the three abiotic stresses (Ag^+^, cold, salt), AgNPs are also novel abiotic stressors that pose different toxicity risks to *Arabidopsis* plants.

## Supplementary Materials

Supplementary materials can be accessed at: http://www.mdpi.com/2079-4991/5/2/436/s1.
