# Supplementary material for: Gene Expression, Protein Function and Pathways of Arabidopsis thaliana Responding to Silver Nanoparticles in Comparison to Silver Ions, Cold, Salt, Drought, and Heat
Source: Nanomaterials (Basel). 2015 Mar 27;5(2):436–67. doi: 10.3390/nano5020436 (PMC5312895; doi:10.3390/nano5020436)
Supplement: Supplementary file 1 [file nanomaterials-05-00436-s001.zip › Supplementary documents/Figure S1.pdf]

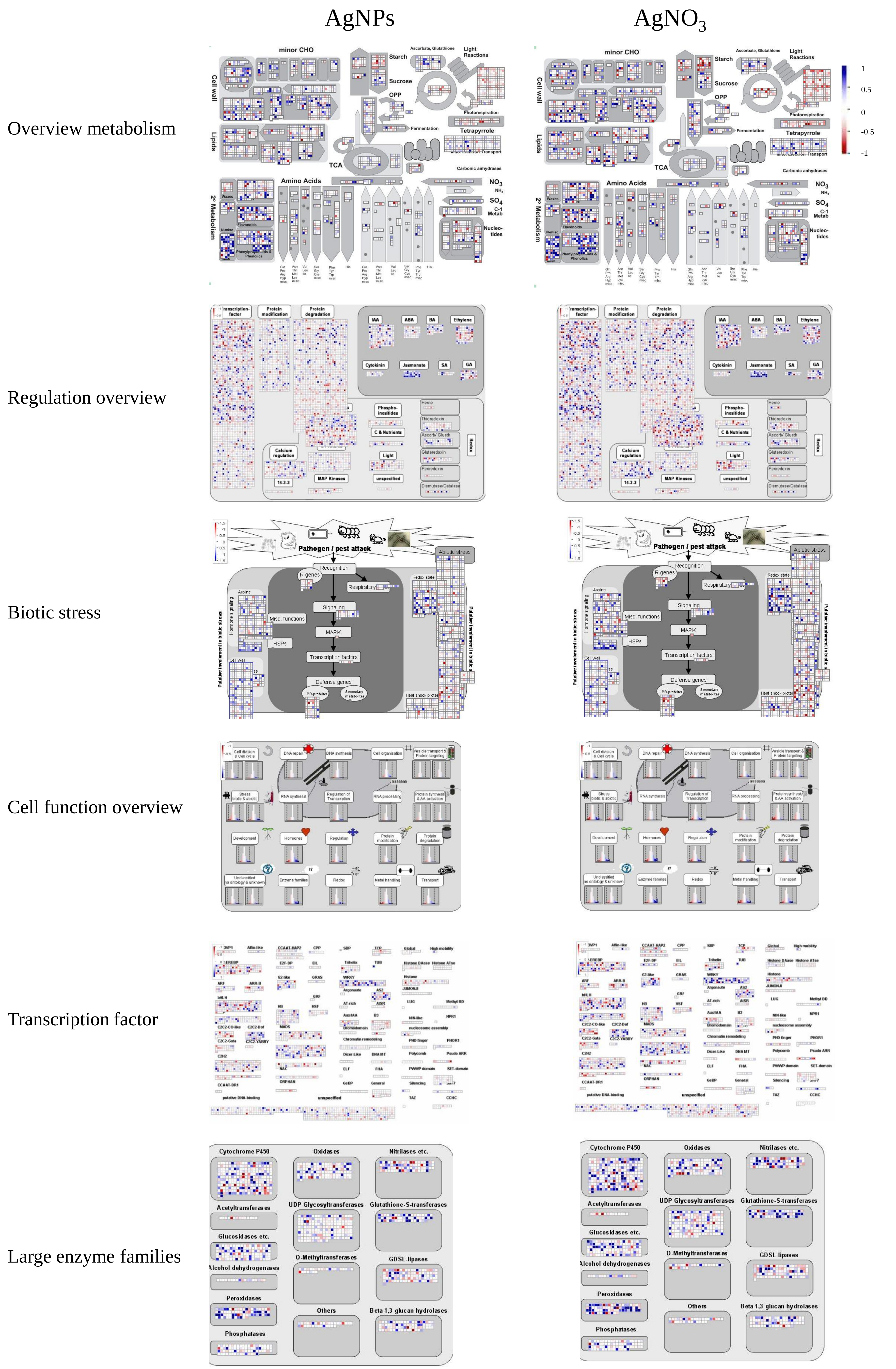

AgNPs
AgNO3
Overview metabolism
Regulation overview
Biotic stress
Cell function overview
Transcription factor
Large enzyme families

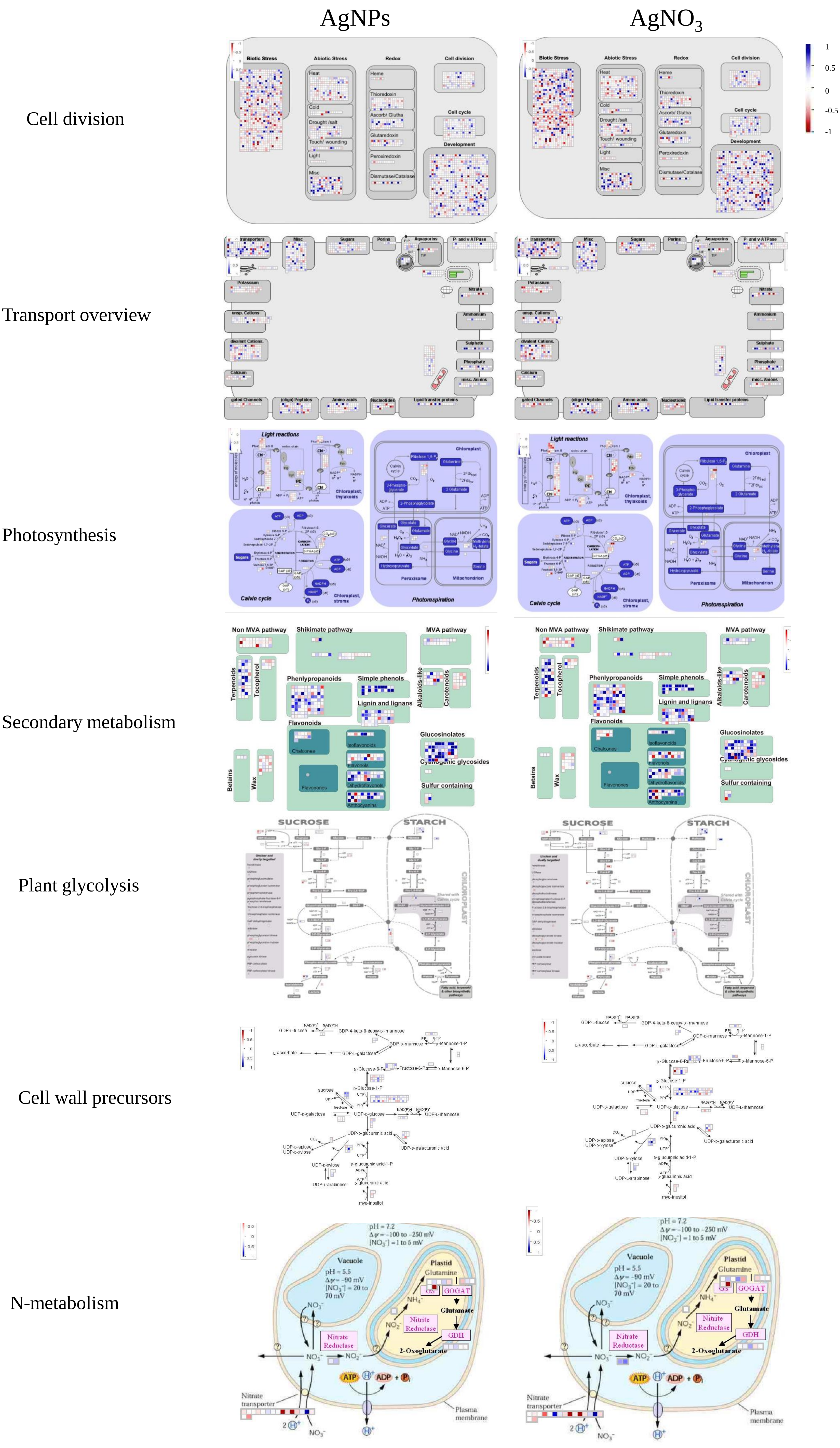

AgNPs
AgNO3
Cell division
Transport overview
Photosynthesis
Secondary metabolism
Plant glycolysis
Cell wall precursors
N-metabolism
1
0.5
0
-0.5
-1
Biotic Stress
Abiotic Stress
Redox
Cell division
Cell cycle
Development
Non MVA pathway
Shikimate pathway
MVA pathway
Phenlypropanoids
Simple phenols
Lignin and lignans
Flavonoids
Glucosinolates
Sulfur containing
SUCROSE
STARCH
CHLOROPLAST
Vacuole
Plastid
Glutamine
Glutamate
2-Oxoglutarate
Nitrate Reductase
Nitrite Reductase
GS
GOGAT
GDH
Nitrate transporter
Plasma membrane

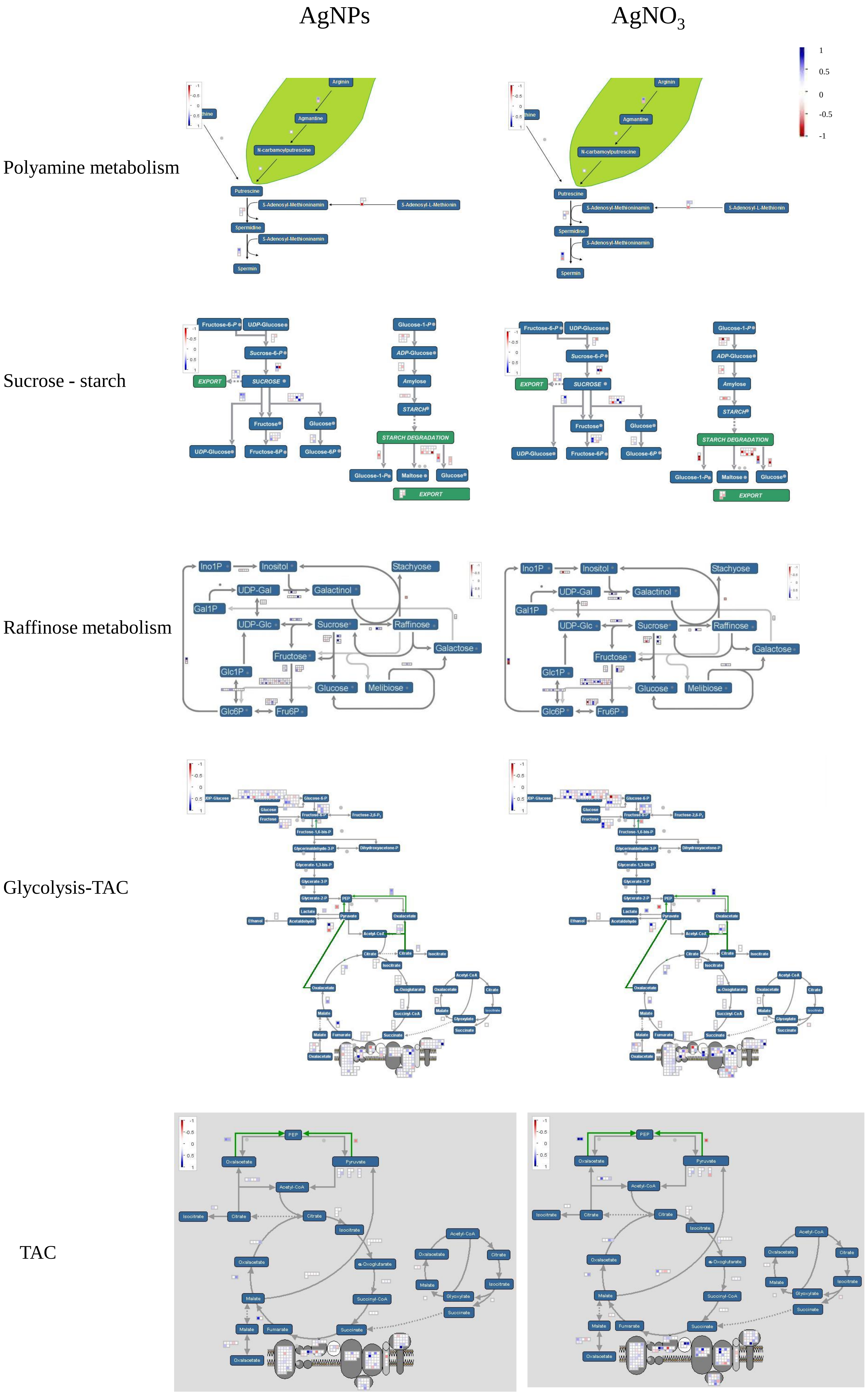
AgNPs
AgNO3
1
0.5
0
-0.5
-1
Polyamine metabolism
Sucrose - starch
Raffinose metabolism
Glycolysis-TAC
TAC

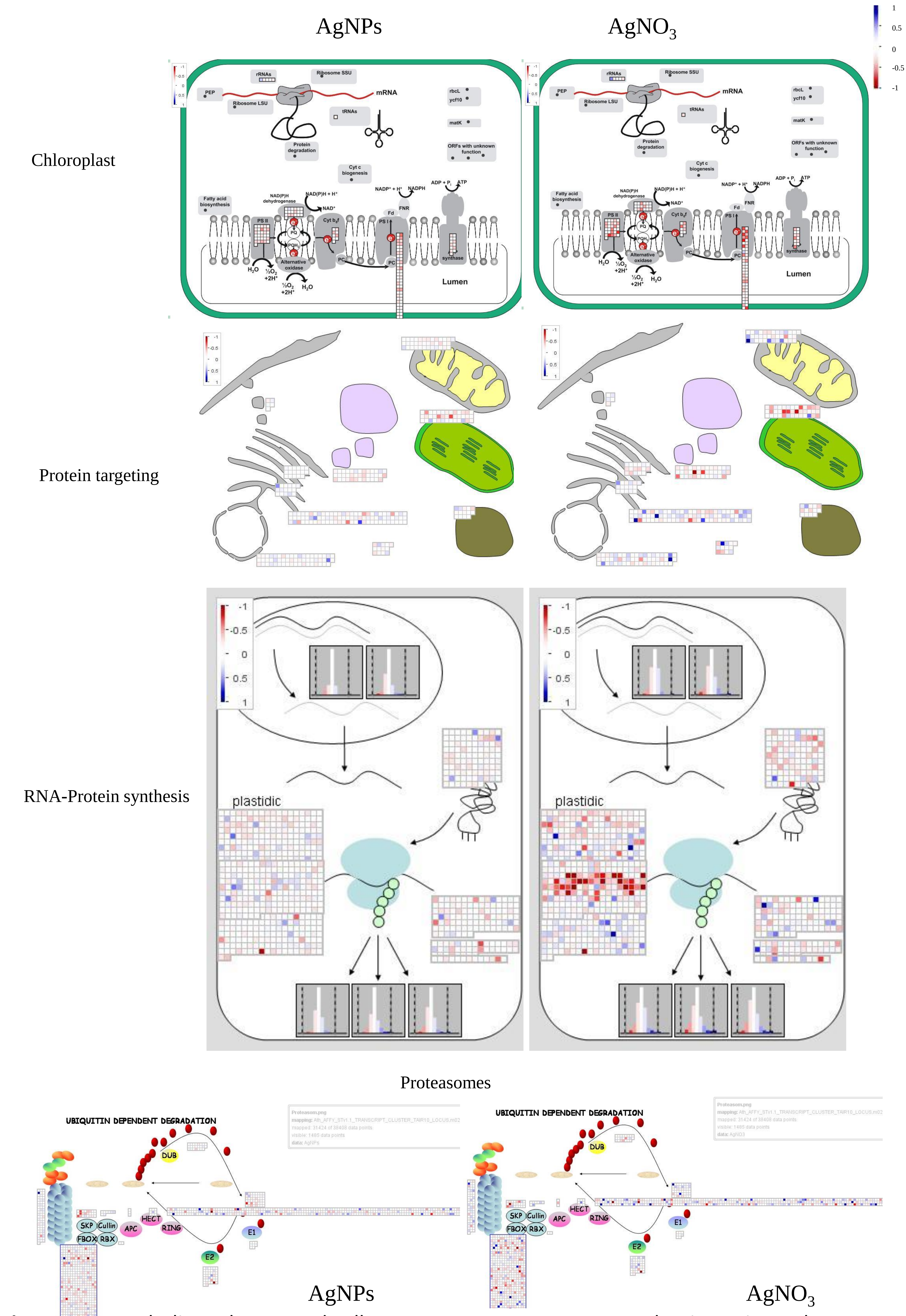

Figure S1. Metabolic pathways and cell compartments were compared to investigate the differences between AgNP with Ag^{+} stresses.
