# Supplementary material for: Gene Expression, Protein Function and Pathways of Arabidopsis thaliana Responding to Silver Nanoparticles in Comparison to Silver Ions, Cold, Salt, Drought, and Heat
Source: Nanomaterials (Basel). 2015 Mar 27;5(2):436–67. doi: 10.3390/nano5020436 (PMC5312895; doi:10.3390/nano5020436)
Supplement: Supplementary file 1 [file nanomaterials-05-00436-s001.zip › Supplementary documents/Figures S2_S7.pdf]

a

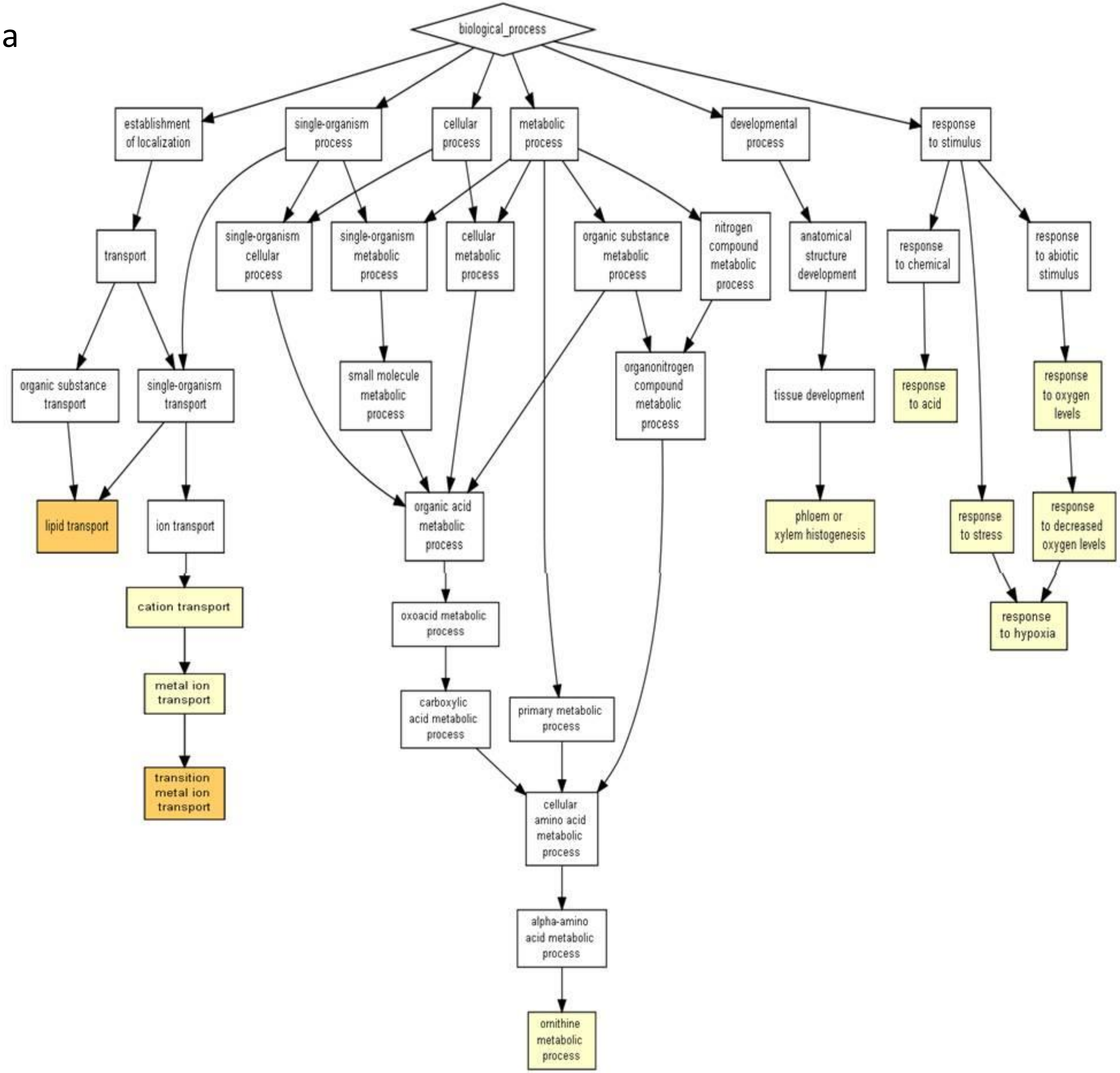

biological_process
establishment of localization
single-organism process
cellular process
metabolic process
developmental process
response to stimulus
transport
single-organism cellular process
single-organism metabolic process
cellular metabolic process
organic substance metabolic process
nitrogen compound metabolic process
anatomical structure development
response to chemical
response to abiotic stimulus
organic substance transport
single-organism transport
small molecule metabolic process
organonitrogen compound metabolic process
tissue development
response to acid
response to oxygen levels
lipid transport
ion transport
organic acid metabolic process
phloem or xylem histogenesis
response to stress
response to decreased oxygen levels
cation transport
oxoacid metabolic process
response to hypoxia
metal ion transport
carboxylic acid metabolic process
primary metabolic process
transition metal ion transport
cellular amino acid metabolic process
alpha-amino acid metabolic process
ornithine metabolic process

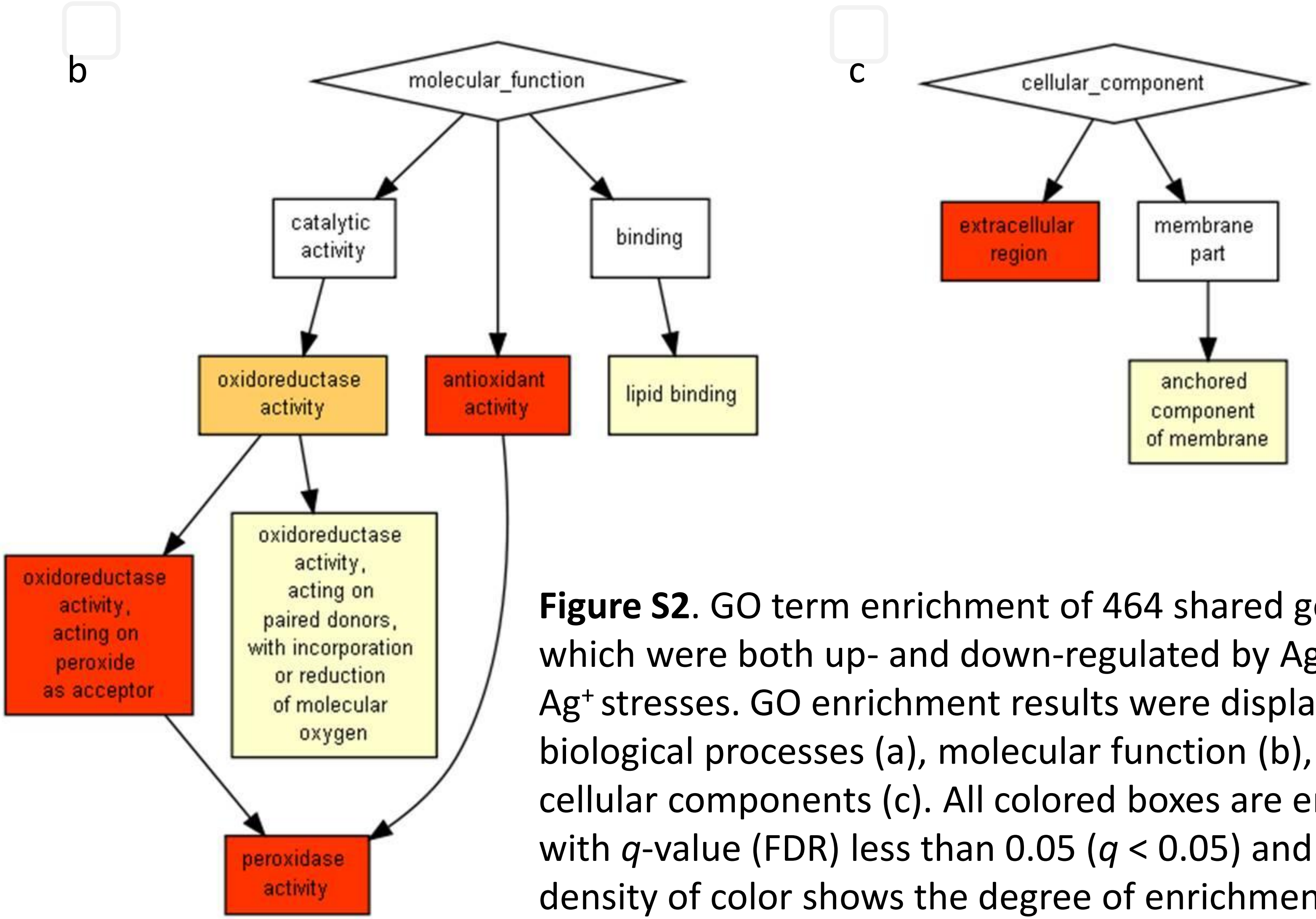

Figure S2. GO term enrichment of 464 shared genes which were both up- and down-regulated by AgNP and Ag^+ stresses. GO enrichment results were displayed for biological processes (a), molecular function (b), and cellular components (c). All colored boxes are enriched with *q*-value (FDR) less than 0.05 ($q < 0.05$) and the density of color shows the degree of enrichment, i.e. red (*p*-value $< 10^{-9}$) > dark orange (*p* -value between 10^{-7} and 10^{-9}) > orange (*p* -value between 10^{-5} and 10^{-7}) > yellow (*p* -value between 10^{-3} and 10^{-5}).

a

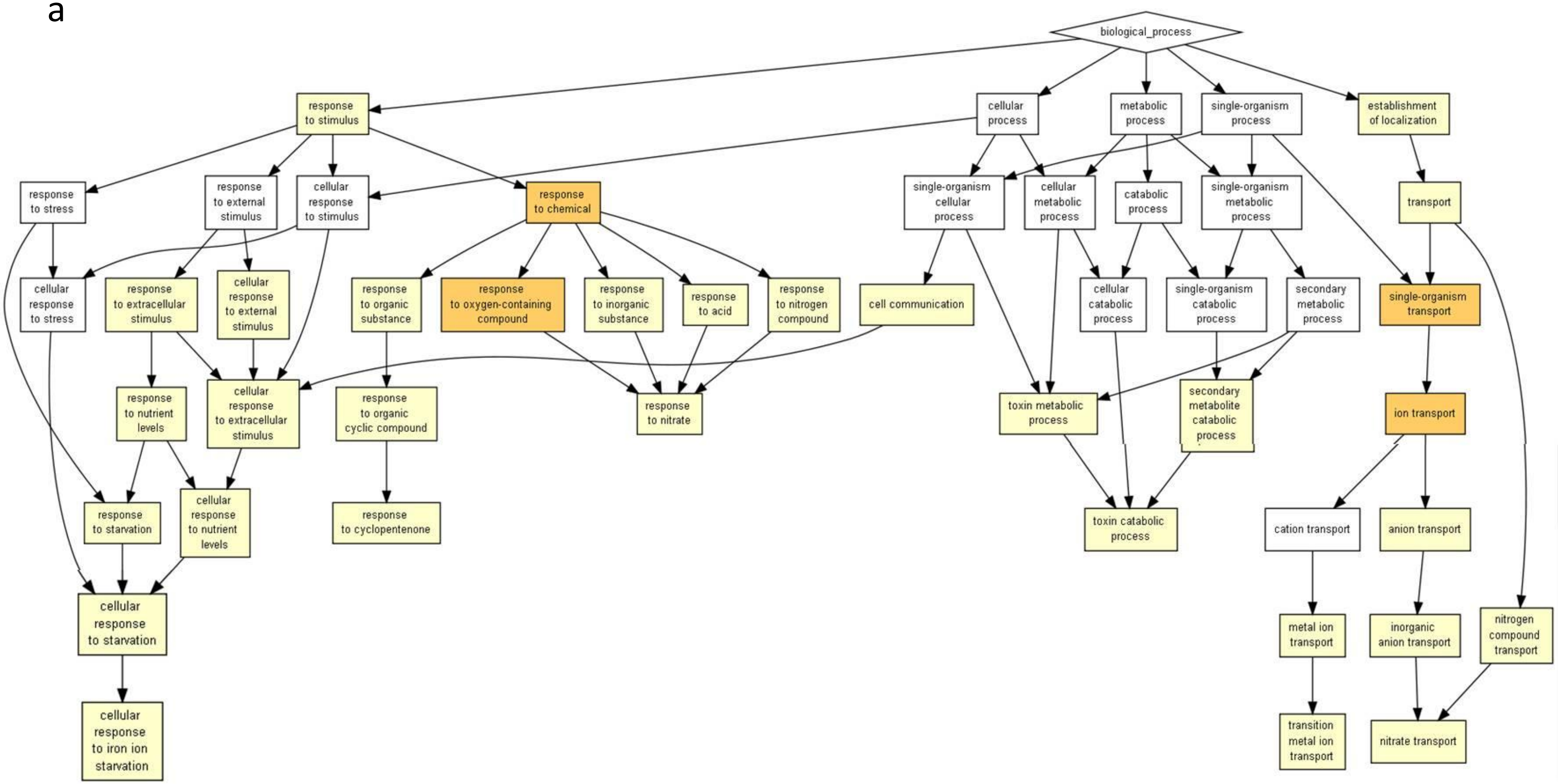

biological_process
response to stimulus
cellular process
metabolic process
single-organism process
establishment of localization
response to stress
response to external stimulus
cellular response to stimulus
response to chemical
single-organism cellular process
cellular metabolic process
catabolic process
single-organism metabolic process
transport
cellular response to stress
response to extracellular stimulus
cellular response to external stimulus
response to organic substance
response to oxygen-containing compound
response to inorganic substance
response to acid
response to nitrogen compound
cell communication
cellular catabolic process
single-organism catabolic process
secondary metabolic process
single-organism transport
response to nutrient levels
cellular response to extracellular stimulus
response to organic cyclic compound
response to nitrate
toxin metabolic process
secondary metabolite catabolic process
ion transport
response to starvation
cellular response to nutrient levels
response to cyclopentenone
toxin catabolic process
cation transport
anion transport
cellular response to starvation
metal ion transport
inorganic anion transport
nitrogen compound transport
cellular response to iron ion starvation
transition metal ion transport
nitrate transport

b

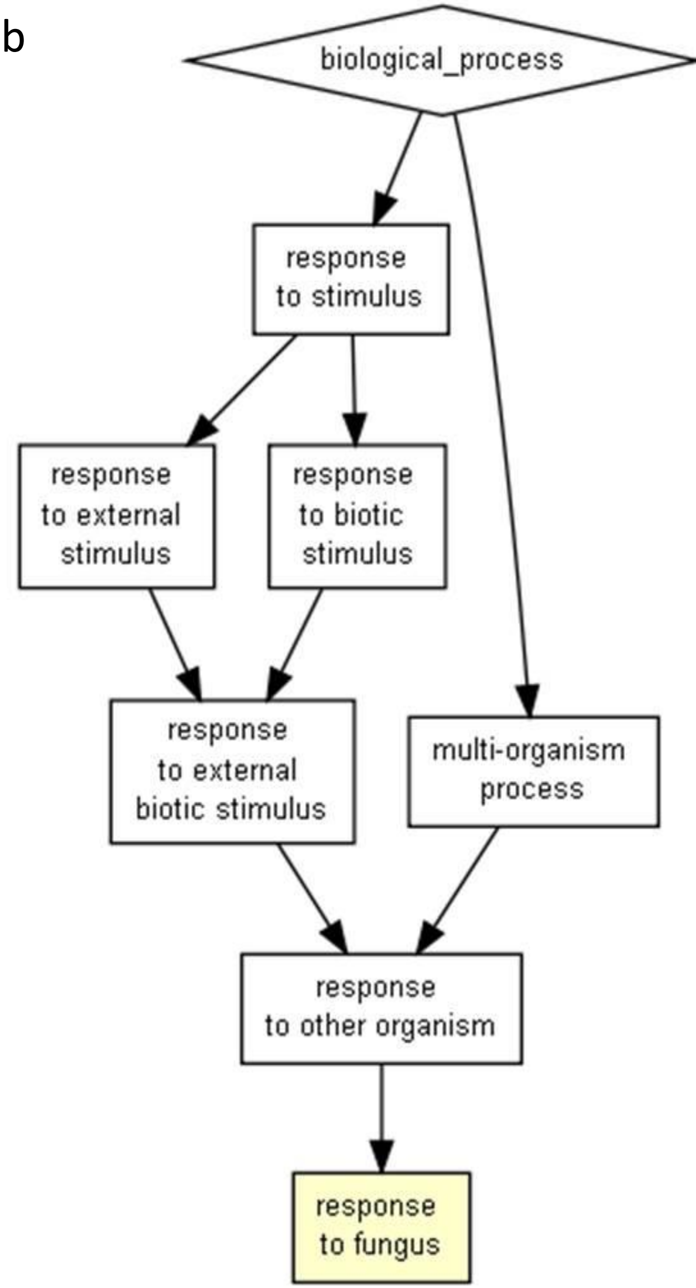

Figure S3. GO term enrichment of AgNP- and Ag^{+}-specific genes. (a) GO enrichment result in the biological processes for 546 Ag^{+} specific genes. (b) GO enrichment result in the biological processes for 111 AgNP specific genes. There was no enrichment in the molecular function and the cellular components for AgNP- and Ag^{+}-specific genes. All colored boxes are enriched with *q*-value (FDR) less than 0.05 ($q < 0.05$) and the density of color shows the degree of enrichment, i.e. red (*p*-value $< 10^{-9}$) > dark orange (*p*-value 10^{-7} to 10^{-9}) > orange (*p*-value 10^{-5} to 10^{-7}) > yellow (*p*-value 10^{-3} to 10^{-5}) > white (*p*-value $> 10^{-3}$).

a

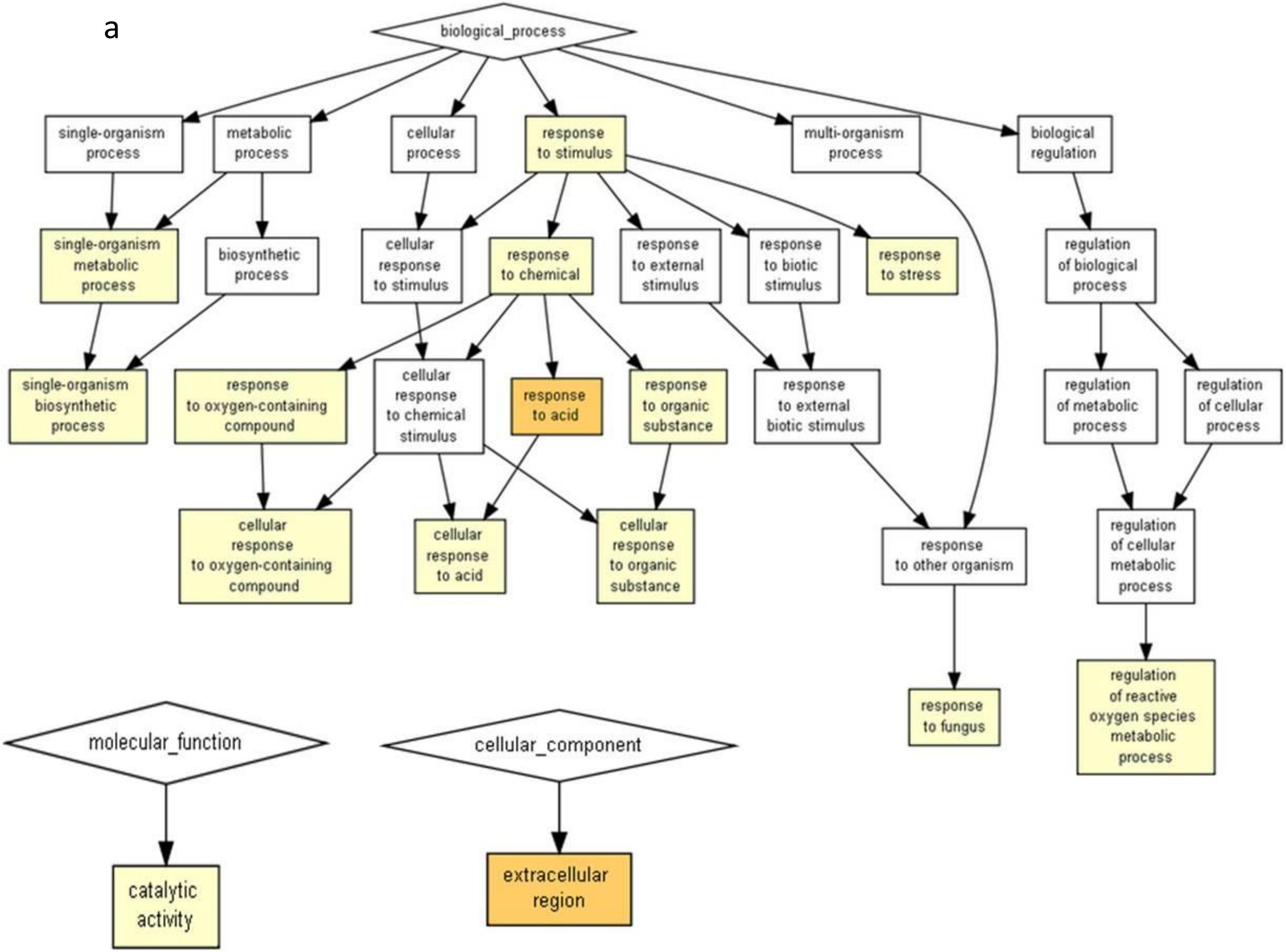

biological_process
single-organism process
metabolic process
cellular process
response to stimulus
multi-organism process
biological regulation
single-organism metabolic process
biosynthetic process
cellular response to stimulus
response to chemical
response to external stimulus
response to biotic stimulus
response to stress
regulation of biological process
single-organism biosynthetic process
response to oxygen-containing compound
cellular response to chemical stimulus
response to acid
response to organic substance
response to external biotic stimulus
regulation of metabolic process
regulation of cellular process
cellular response to oxygen-containing compound
cellular response to acid
cellular response to organic substance
response to other organism
regulation of cellular metabolic process
response to fungus
regulation of reactive oxygen species metabolic process
molecular_function
catalytic activity
cellular_component
extracellular region

b c

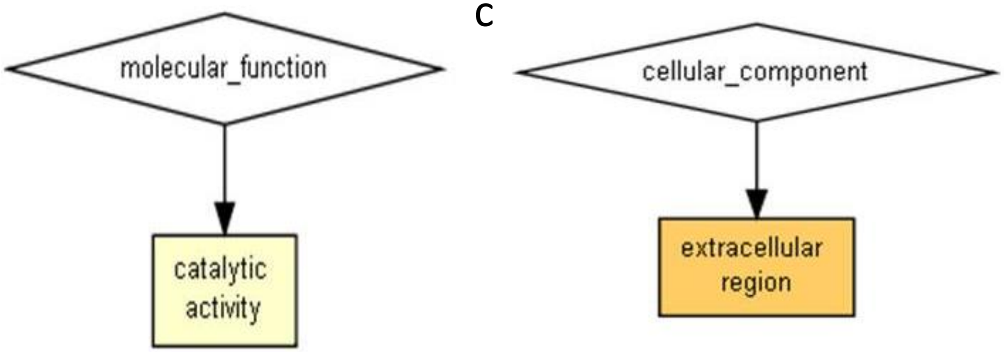

Figure S4. GO term enrichment of 202 AgNP and cold shared genes. GO enrichment results were display for biological processes (a), molecular function (b), and cellular components (c). All colored boxes were enriched with *q*-value (FDR) less than 0.05 ($q < 0.05$) and the density of color shows the degree of enrichment, i.e. red (*p*-value < 10^{-9}) > dark orange (*p*-value 10^{-7} to 10^{-9}) > orange (*p*-value 10^{-5} to 10^{-7}) > yellow (*p*-value 10^{-3} to 10^{-5}) > white (*p*-value > 10^{-3}).

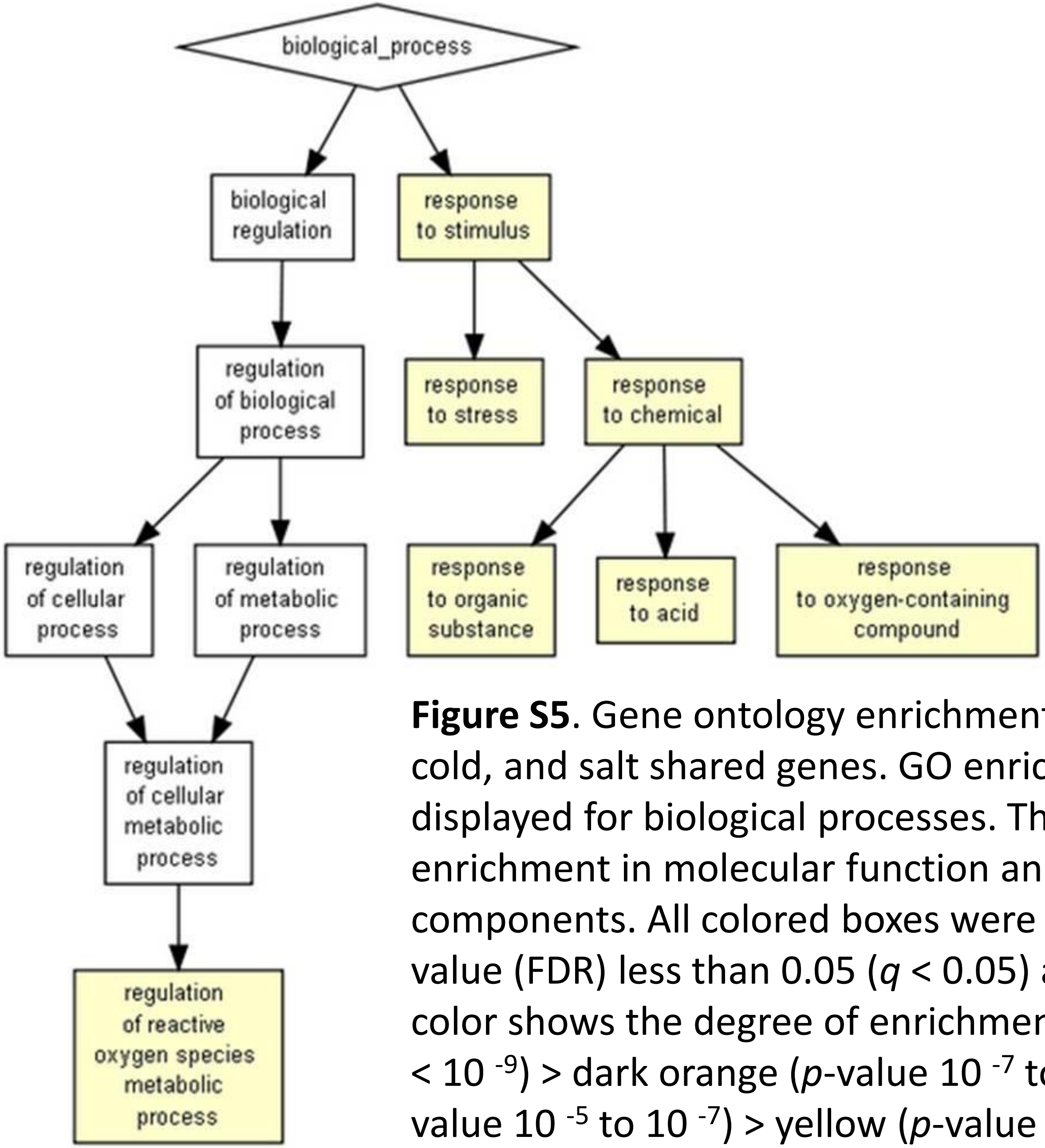

Figure S5. Gene ontology enrichment of 66 AgNP, Ag^+, cold, and salt shared genes. GO enrichment result were displayed for biological processes. There was no enrichment in molecular function and cellular components. All colored boxes were enriched with *q*-value (FDR) less than 0.05 ($q < 0.05$) and the density of color shows the degree of enrichment, i.e. red (*p*-value $< 10^{-9}$) > dark orange (*p*-value 10^{-7} to 10^{-9}) > orange (*p*-value 10^{-5} to 10^{-7}) > yellow (*p*-value 10^{-3} to 10^{-5}) > white (*p*-value $> 10^{-3}$).

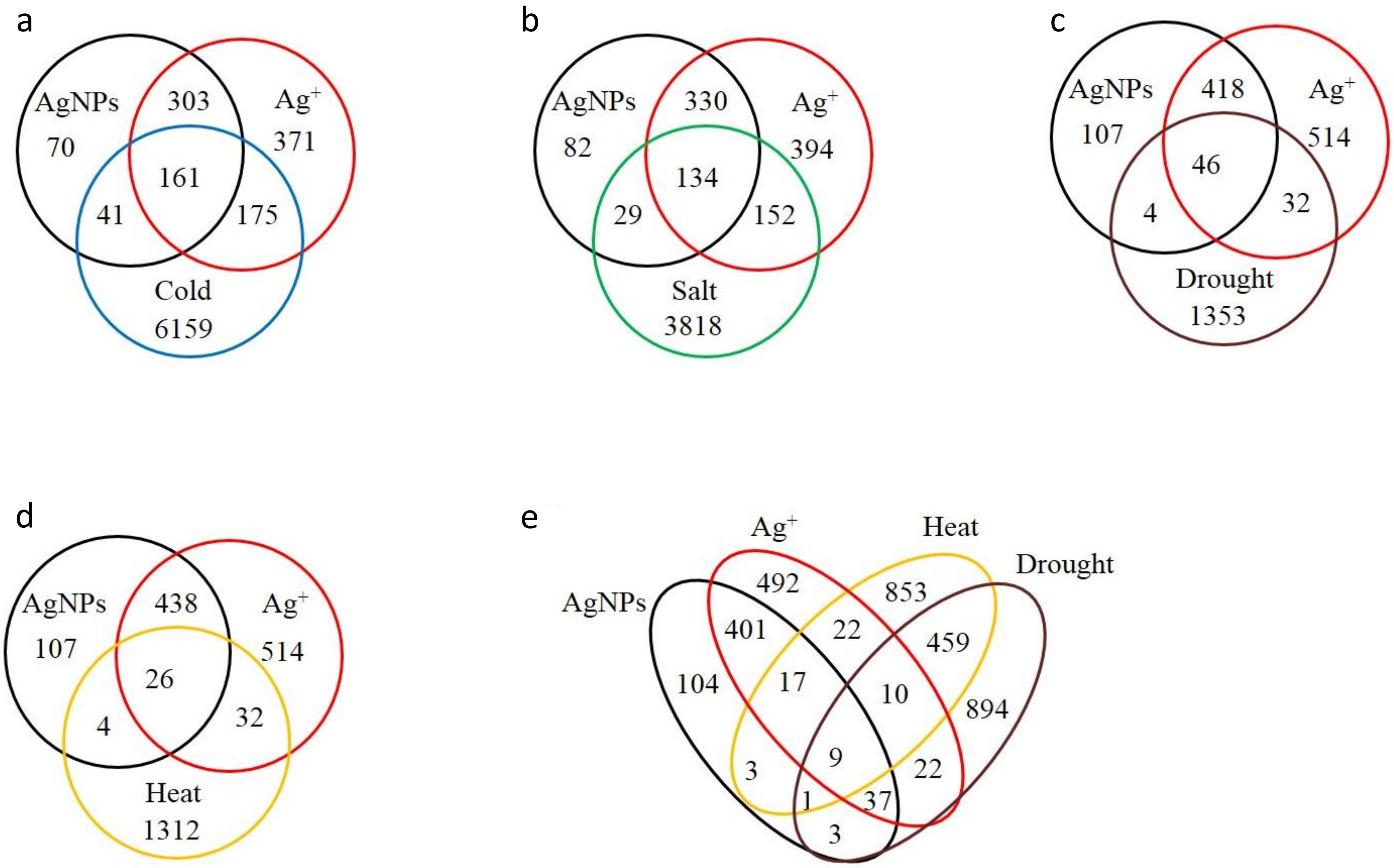

Figure S6. Three and four way Venn diagrams of the genes with more than two fold expression changes among the six stresses. (a) Number of shared and specific genes for AgNPs, Ag^+, and cold. (b) Number of shared and specific genes for AgNPs, Ag^+, and salt. (c) Number of shared and specific genes for AgNPs, Ag^+, and drought. (d) Number of shared and specific genes for AgNPs, Ag^+, and heat. (e) A four-way Venn diagram for shared genes and specific genes in AgNP, Ag^+, drought and heat stresses.

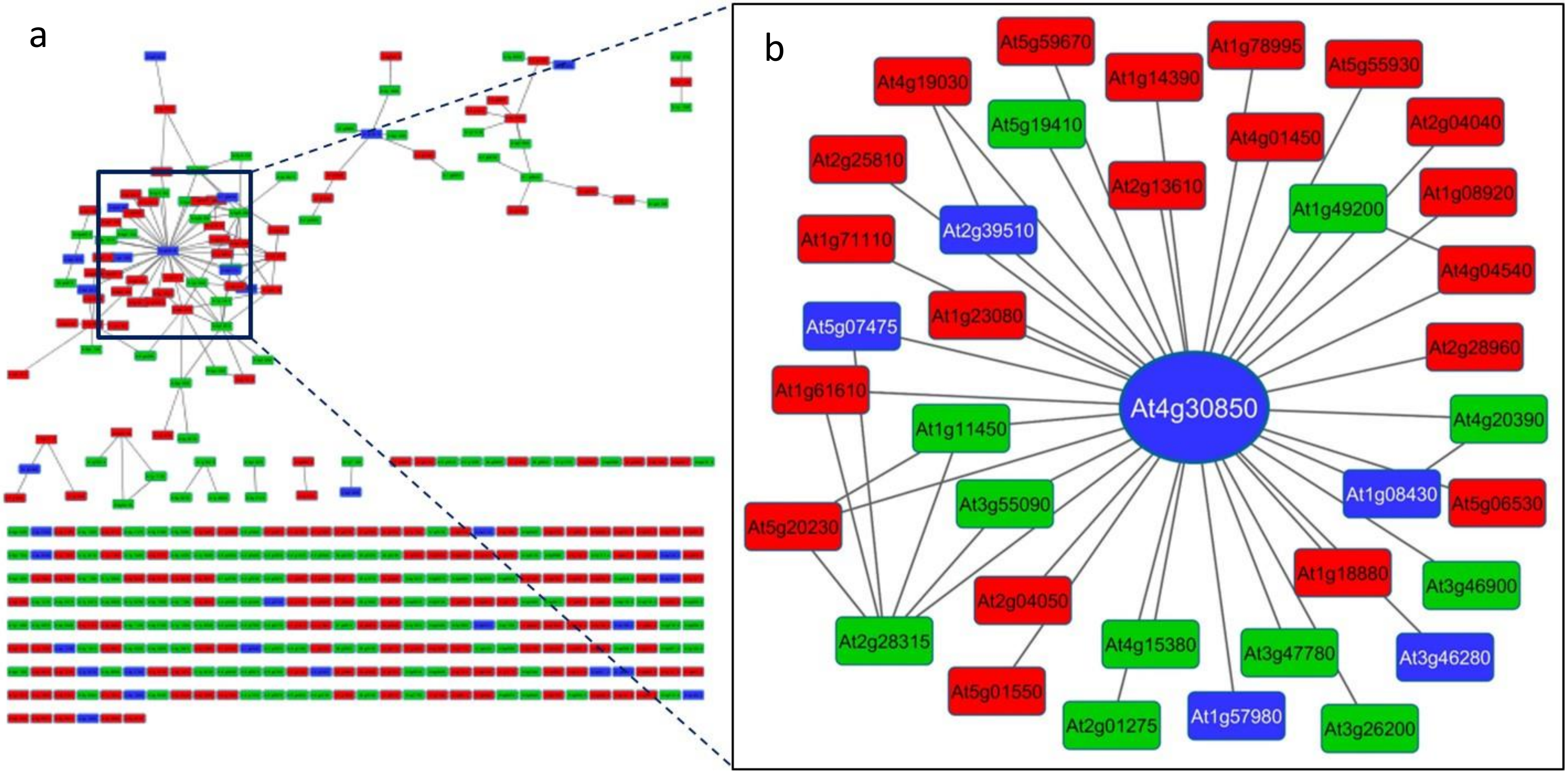

Figure S7. The protein-protein interaction (PPi) network of shared and specific genes related to AgNPs and Ag^+. (a) PPi network of all the gene-encoded proteins affected by AgNPs and Ag^+ in three colors: blue denoted AgNP specific genes, red Ag^+ specific genes, and green AgNPs and Ag^+ shared genes. (b) Broadup of the PPi subnetwork of HHP2 (heptahelical transmembrane protein) as the hub. HHP2 is encoded by At4g30850 gene. At4g30850 gene was up-regulated by AgNPs and HHP2, and its encoded protein HHP2 had the highest connectivity.
